# Lack of astrocytes hinders parenchymal oligodendrocyte precursor cells from reaching a myelinating state in osmolyte-induced demyelination

**DOI:** 10.1186/s40478-020-01105-2

**Published:** 2020-12-24

**Authors:** Melanie Lohrberg, Anne Winkler, Jonas Franz, Franziska van der Meer, Torben Ruhwedel, Nikoloz Sirmpilatze, Rakshit Dadarwal, Ronja Handwerker, Daniel Esser, Kerstin Wiegand, Christian Hagel, Andreas Gocht, Fatima Barbara König, Susann Boretius, Wiebke Möbius, Christine Stadelmann, Alonso Barrantes-Freer

**Affiliations:** 1 Institute of Neuropathology, University Medical Center Göttingen, 37075 Göttingen, Germany; 2 Electron Microscopy Core Unit, Department of Neurogenetics, Max Planck Institute of Experimental Medicine, Göttingen, Germany; 3 Functional Imaging Laboratory, German Primate Center, Leibniz Institute for Primate Research, Göttingen, Germany; 4 Department of Ecosystem Modelling, Georg-August University, Göttingen, Germany; 5 Institute of Neuropathology, University Medical Center Hamburg-Eppendorf, Hamburg, Germany; 6 Institute of Pathology, University Hospital Schleswig-Holstein, Lübeck, Germany; 7 Institute of Anatomy and Experimental Morphology, University Medical Center Hamburg-Eppendorf, Hamburg, Germany; 8 Institute of Pathology, Medical Center, Kassel, Germany; 9 Center for Nanoscale Microscopy and Molecular Physiology of the Brain (CNMPB), Göttingen, Germany; 10 Department of Neuropathology, University Medical Center Leipzig, Leipzig, Germany; 11 Campus Institute for Dynamics of Biological Networks, University of Göttingen, Göttingen, Germany; 12 Max Planck Institute for Experimental Medicine, Göttingen, Germany

**Keywords:** Remyelination, Oligodendrocyte precursor cells, Neural stem cells, Multiple sclerosis, Astrocytes

## Abstract

Demyelinated lesions in human pons observed after osmotic shifts in serum have been referred to as central pontine myelinolysis (CPM). Astrocytic damage, which is prominent in neuroinflammatory diseases like neuromyelitis optica (NMO) and multiple sclerosis (MS), is considered the primary event during formation of CPM lesions. Although more data on the effects of astrocyte-derived factors on oligodendrocyte precursor cells (OPCs) and remyelination are emerging, still little is known about remyelination of lesions with primary astrocytic loss. In autopsy tissue from patients with CPM as well as in an experimental model, we were able to characterize OPC activation and differentiation. Injections of the thymidine-analogue BrdU traced the maturation of OPCs activated in early astrocyte-depleted lesions. We observed rapid activation of the parenchymal NG2^+^ OPC reservoir in experimental astrocyte-depleted demyelinated lesions, leading to extensive OPC proliferation. One week after lesion initiation, most parenchyma-derived OPCs expressed breast carcinoma amplified sequence-1 (BCAS1), indicating the transition into a pre-myelinating state. Cells derived from this early parenchymal response often presented a dysfunctional morphology with condensed cytoplasm and few extending processes, and were only sparsely detected among myelin-producing or mature oligodendrocytes. Correspondingly, early stages of human CPM lesions also showed reduced astrocyte numbers and non-myelinating BCAS1^+^ oligodendrocytes with dysfunctional morphology. In the rat model, neural stem cells (NSCs) located in the subventricular zone (SVZ) were activated while the lesion was already partially repopulated with OPCs, giving rise to nestin^+^ progenitors that generated oligodendroglial lineage cells in the lesion, which was successively repopulated with astrocytes and remyelinated. These nestin^+^ stem cell-derived progenitors were absent in human CPM cases, which may have contributed to the inefficient lesion repair. The present study points to the importance of astrocyte-oligodendrocyte interactions for remyelination, highlighting the necessity to further determine the impact of astrocyte dysfunction on remyelination inefficiency in demyelinating disorders including MS.

**Electronic supplementary material:**

The online version of this article (10.1186/s40478-020-01105-2) contains supplementary material, which is available to authorized users

## Introduction

Several diseases of the central nervous system are characterized by more or less selective damage to the myelin sheaths, referred to as demyelination. A common aspect of all demyelinating diseases (including their respective experimental models) is the inherent capacity of the adult brain to react to myelin loss by a spontaneous regeneration process, termed remyelination. In multiple sclerosis (MS) patients, a higher degree of remyelination has been related to less disability [9, 12]. But especially in MS, remyelination is highly variable and becomes less efficient with age [12, 13, 29, 34, 53, 69, 70, 73, 80]. Oligodendrocyte precursor cells (OPCs) are crucial for remyelination because they are able to differentiate into new oligodendrocytes, thereby enwrapping axons with myelin [18, 19, 77, 96, 107]. Still, current studies in animal models as well as in MS patients point to a remyelinating capacity of preexisting and probably also mature oligodendrocytes [24, 106].

Myelination is most efficient during development. Under pathological conditions, remyelination is inherently limited, as most demyelinating lesions are in an environment very different from the healthy developing brain [28]. As the key players in myelination, oligodendrocytes are in close contact to other glial cells, particularly astrocytes. All processes necessary for myelination such as recruitment, proliferation, differentiation and maturation of oligodendrocytes, are tightly regulated by factors secreted by astrocytes or by direct cell-to-cell contact (see review [23]). Astrocytic impairment in the context of myelin loss and inflammation is observed in several demyelinating diseases, particularly in neuromyelitis optica (NMO) as well as in progressive multifocal leukoencephalopathy (PML). Astrocytic loss has also recently been reported in acute MS lesions [74]. However, the role of astrocytes in demyelinating diseases is still not fully understood, with contradictory reports claiming either beneficial or detrimental effects [2, 3, 8, 27, 35, 85, 86, 88, 95].

Primary astrocytic loss followed by secondary oligodendrocyte and myelin loss is rare, but has been reported in Alexander disease, which is caused by a mutation in the GFAP gene [90] and in NMO due to autoantibodies targeting the astrocytic water channel aquaporin-4 (AQP4) [48, 56, 103]. Moreover, osmotic insults have been connected to primary astrocytic loss, leading to the osmotic demyelinating syndrome (ODS) [1, 30].

ODS is most often linked to rapid correction of chronic hyponatremia, which leads to demyelinated lesions in the pons (central pontine myelinolysis, CPM) or other CNS regions (extrapontine myelinolysis, EPM) [1, 40, 43, 51, 66, 87, 93, 102]. ODS is most often accompanied by comorbidities that include shifts in Na^+^ levels, e.g. alcoholism or liver transplantation (see reviews [45, 65]). The main histological characteristics of ODS, besides demyelination, are reduced astrocyte densities with loss of aquaporins, widespread macrophage/microglia activation with myelin phagocytosis and relative neuronal and axonal preservation despite acute axonal damage early in lesion formation [33, 72]. There is little data on remyelination in ODS; only one post-mortem case report with histologically confirmed remyelination in an asymptomatic CPM patient, analyzed 2 years after MRI-based diagnosis, is available in the literature [36]. Studying remyelination in lesions with primary astrocyte pathology can provide important insights into the mechanisms of CNS regeneration of pathologies generally associated with severe clinical deficits and poor myelin repair such as MS.

Experimental models mimicking histopathological characteristics of human CPM lesions have been established in several species [11, 43, 68]. Using these models, astrocyte pathology has been confirmed as the primary event in lesion formation [30]. Consequently, first indications of myelin loss can be seen 24–48 h after astrocytic loss, followed by microglia activation [30, 43]. Disturbance of the panglial syncytium, as indicated by a downregulation of gap junction proteins Cx43 and Cx47 at the beginning of astrocyte pathology, is hypothesized to lead to the loss of oligodendrocytes [11, 30]. In ODS mice, adenomatous polyposis coli (APC)-positive oligodendrocytes are nearly absent prior to myelin loss but gradually return to the lesion site afterwards. Thus, a secondary demyelination following the demise of oligodendrocytes as opposed to a direct osmotic insult to the myelin sheaths, can be assumed [11].

The present study aims at investigating the cellular mechanisms of remyelination in the presence of a prominent primary astrocytopathy. We focus particularly on the recruitment of parenchymal versus stem cell-derived oligodendrocytes and the temporal relation to astrocytic repopulation.

## Methods

### Human tissue

This retrospective study was performed in accordance with the ethical standards as laid down in the 1964 Declaration of Helsinki and its later amendments, and was approved by the institutional ethics committee of the University Medical Center Göttingen. To characterize remyelination and further neuropathological characteristics of CPM, microscopic transverse sections of the pons from archival autopsied tissue from the University Medical Center Göttingen (Germany) and the University Medical Center Hamburg-Eppendorf (Germany) were used. 18 pathologically confirmed CPM and 8 control cases were included. Inclusion criteria were the presence of recognizable demyelinating lesions in the pons upon LFB-PAS staining and sufficient archival tissue for pathological analysis. In addition, for anti-nestin immunohistochemistry (IHC) archival autopsy tissue of fetal pons was used. Clinical information was obtained from the medical records (Additional file 1: Table S1). Clinical and histopathological information on 7 cases has been previously published [32]. CPM patients had a mean age of 50.3 ± 19.7 years, control patients 58.6 ± 15.2 years. A history of alcohol abuse was recorded for 15/18 CPM cases.

Light and fluorescence IHC on paraffin sections of human autopsy tissue were carried out according to standard procedures using the antibodies listed in Table 1.

**Table 1:** Primary antibodies I (human tissue)

Antibody	Species	Dilution	Antigen retrieval	Manufacturer
Anti-AQP4	Rabbit	1:200	Citrate, MW	Sigma Aldrich, A5971
Anti-BCAS1	Rabbit	1:100	Citrate, MW	Abcam, ab106661
Anti-GFAP	Rabbit	1:1000	–	Dako, Z0334
Anti-KiM1P	Mouse	1:5000	Citrate, MW	Radzun et al. [76]
Anti-MAG	Mouse	1:5000	Citrate, MW	Abcam, ab89780
Anti-Olig2	Mouse	1:50	EDTA, MW	Merck Millipore, MABN50
Anti-TPPP/p25	Rabbit	1:500	EDTA, MW	Abcam, ab92305
Anti-Sox10	Mouse	1:100	Citrate, MW	Novus, NBP2-59620

### Animals

This study was carried out at the University Medical Center Göttingen in strict accordance with recommendations of European and German guidelines for welfare of experimental animals. Animal experiments were approved by the Review Board for the Care of Animal Subjects of the district government of Lower Saxony (LAVES, approval number 13/1197). Female Lewis rats (Charles River Laboratories), aged approximately 3 months, were included in the study. Unless specified otherwise, anesthesia was performed using i.p. injection of ketamine (60 mg/kg) and xylazine (8 mg/kg).

### ODS protocol

ODS was induced according to an adapted protocol [98]. Briefly, an osmotic minipump (Model 1007D, Charles River Laboratories, Germany) filled with desmopressin (dDAVP, 10 µg/ml, Sigma Aldrich, USA) was implanted subcutaneously caudal to the shoulder blade (day 0). Standard chow was switched to low-sodium liquid diet (EF15710-10 EF R/M AIN 76A, Ssniff, Germany), fed ad libitum during hyponatremia. At day 6, i.p. injection of sodium chloride solution (1 M, 1 ml per 100 g body weight) was used to increase serum sodium levels close to normonatremia. After sodium correction, food was switched back to standard pellet chow. At days 0 and 6, blood sodium levels were measured. All animals were monitored daily and brain tissue was harvested after 3 (n = 4), 4 (n = 3), 6 (n = 4), 7 (n = 3), 13 (n = 5), 14 (n = 2) and 21 (n = 6) days post correction (dpc). Untreated age-matched rats were used as healthy controls (n = 5).

### Bromodeoxyuridine (BrdU) labelling

In an additional subset of 9 animals, the thymidine analogue BrdU was injected to mark proliferating cells. ODS was induced as described above. Additionally, from day 1–3 after correction of hyponatremia, isotonic NaCl solution containing 100 mg/kg bodyweight BrdU (Sigma Aldrich, USA) was injected every 12 h. Brain tissue was harvested for analysis 4, 7, 14 and 21 days after correction (4dpc n = 1, 7dpc n = 3, 14dpc n = 3, 21dpc n = 2). Four control animals without induction of ODS were injected with BrdU, and tissue from one animal was harvested at days 4, 7, 14 and 21, respectively.

### Histology: Animals

Rats were anesthetized and transcardially perfused with phosphate-buffered paraformaldehyde (PFA, 4%). Brains were removed and further processed according to standard protocols. Formalin-fixed and paraffin-embedded brain tissue was sectioned into 2–3 µm thick sections. Histochemical, immunohistochemical and immunofluorescent techniques were applied using standard protocols. Primary antibodies are listed in Table 2. For immunofluorescence multi-labeling, Tyramide SuperBoost™ kits (Invitrogen) were used.

**Table 2:** Primary antibodies II (rat tissue)

Antibody	Species	Dilution	Antigen retrieval	Manufacturer
Anti-BCAS1 (Anti-NABC1)	Rabbit	1:100	Citrate, MW	Abcam, ab106661
Anti-BrdU	Mouse	1:400	Citrate, MW	Millipore, MAB3424
Anti-CD68 (clone ED1)	Mouse	1:500	Citrate, MW	Biorad, MCA341R
Anti-GFAP	Rat	1:1000	Citrate, MW	Thermo Fisher, Z0334
Anti-Ki67	Rabbit	1:200	Citrate, MW	DCS, K1681C01
Anti-MAG	Mouse	1:1000	Citrate, MW	Abcam, ab89780
Anti-MBP	Rabbit	1:500	–	Dako, A0623
Anti-Nestin	Mouse	1:50	Citrate, MW	Abcam, ab6142
Anti-NG2	Rabbit	1:200	EDTA, MW	Millipore, AB5320
Anti-Olig2	Mouse	1:100	EDTA, MW	Millipore, MABN50
Anti-Olig2	Rabbit	1:50	EDTA, MW	IBL, 18953
Anti-TPPP/p25	Rabbit	1:500	EDTA, MW	Abcam, ab92305
Anti-PDGFRalpha	Rabbit	1:100	Citrate, MW	Abcam, ab203491

Fluorescent terminal deoxynucleotidyl transferase (TdT) dUTP Nick-End Labeling (TUNEL) assay together with an anti-Olig2 immunofluorescent labeling was performed to detect late stage apoptotic oligodendrocytes with degraded DNA. TUNEL assay was carried out using a kit (Roche #03333574001) according to the manufacturer’s instructions. For fluorescent labeling anti-digoxygenin-rhodamine Fab fragments (Roche #112077339810) were used. Slides were doublestained with anti-Olig2 immunofluorescence labeling (see Table 2).

### Image acquisition and analysis

Brightfield microphotographs of tissue sections were acquired using a light microscope (BX51, Olympus, Tokyo, Japan) equipped with a digital camera (DP71, Software CellSens Dimension v.1.7.1, Olympus, Tokyo, Japan). Immunofluorescence pictures were taken using a fluorescence microscope (BX63, Olympus, Tokyo, Japan) equipped with a digital camera (DP80, CellSens Dimension v.2.3, Olympus, Tokyo, Japan). Post-acquisition processing was done using Adobe Photoshop CS6 software. For analysis, immunofluorescent sections were scanned using a virtual slide scanner (VS120, Olympus, Tokyo, Japan). Lesion areas were manually delineated and measured using ImageJ (FIJI) software [81]. Cell densities were determined by manual counting of cells using the ImageJ cell counter plugin and division through the area considered and given as cells/mm^2^. Graph PadPrism 6.0 was employed for data plotting. For spatial analysis, manually labeled cell coordinates were used. Kernel density maps were estimated with a kernel size estimated by the bandwidth method (bandwidth = 0.075) by using the open source python packages scipy and shapely which interface by GEOS the open source library Java Topology Suite (JTS). Distance plots were calculated for binned intervals of 50 µm and 95% confidence interval were estimated assuming a Gaussian distribution of single measured values.

### Electron microscopy (EM)

In a subset of at least four animals per group, EM analysis of the striatal fibers was carried out for ultrastructural assessment of the extent of demyelination and remyelination. Tissue preparation by high-pressure freezing and tissue embedding was performed as described previously [101]. Briefly, the animal was euthanized, the brain was removed and parasagittal vibratome sections were cut. The samples were then frozen in liquid nitrogen using a high-pressure freezer (approximately 2000 bar, Leica HPM100) and further processed by freeze substitution and embedding in epoxy resin for transmission electron microscopy. In a next step, 50 nm thick sections were cut for EM (EM 10, Zeiss, Germany) and a minimum of 5 images per animal were taken from the lesion area at a magnification of 3150x, using the AnalySIS image processing software 3.2. g-ratios (axon diameter divided by fiber diameter) were calculated for at least 30 fibers per picture. In addition, the percentage of myelinated axons relative to total axon counts was determined. In the graph depicting the percentage of myelinated axons, each point represents one animal, whereas for g-ratios each point represents one axon. Graph PadPrism 6.0 was used for data plotting.

### RT-PCR

To assess relative mRNA expression levels, qPCR of striatal tissue was performed. Total RNA was isolated from fresh brain tissue using the RNeasy Micro Kit (Qiagen). RNA was isolated 3 (n = 5), 7 (n = 5), 14 (n = 3) and 21 (n = 3) dpc from the striatal lesion, as well as from healthy controls (n = 3). The mRNA was transcribed into cDNA using the High Capacity RNA-to-cDNA™ Kit (Life Technologies) according to the manufacturer’s instructions. Furthermore, cDNA was used for qPCR using the qPCR core kit (Eurogentec). The following TaqMan™ primers were obtained from Thermo Fisher Scientific (USA) and used as indicated by manufacturer’s protocol: Olig2 (Rn01767116_m1), MAG (Rn01457782_m1), GAPDH (Rn01775763_g1). Relative expression of oligodendrocyte-specific genes Olig2 and MAG was normalized to mean oligodendrocyte densities at the respective time point.

### Magnetic resonance imaging (MRI) and analysis

To follow ODS lesion progression and repair in vivo high-field MRI (9.4 Tesla, Bruker BioSpin MRI GmbH, Ettlingen, Germany) was performed in one rat at 0 (baseline), 1, 7, 14, and 21 dpc. The animal was initially anesthetized with 5% isoflurane, subsequently intubated and kept under anesthesia with 1.5% isoflurane in oxygen and medical air (1:1). The MRI protocol included T2-weighted images (TURBO-RARE, TR 6275 ms, TE 40 ms, RARE factor 8, 50 consecutive axial slices with 500 µm thickness, in-plane resolution 117 × 117 µm^2^), myelin water imaging (3D multiple spin-echo, TR 5040 ms, TE 6 ms, 20 echoes, echo spacing 6 ms, 40 axial slices with 400 µm thickness and 200 µm slice gap, in-plane resolution 200 × 200 µm^2^) and diffusion-weighted MRI (Stejskal-Tanner pulsed gradient spin-echo, echo planar imaging, TR 2000 ms, TE 21.2 ms, 40 axial slices with 400 µm thickness and 200 µm slice gap, in-plane resolution 200 × 200 µm^2^, b-values 1000 and 2000 s/mm^2^, 30 directions each, 5 b0 images). The T2 signal decay was fitted multi-exponentially to estimate the T2 relaxation times for myelin water and intra/extracellular water (T2IEW), and to calculate the myelin water fraction (MWF) [15, 54, 55]. Diffusion tensor was calculated as described before and parametric maps including fractional anisotropy (FA) and radial diffusivity (RD) were derived [5, 31]. Based on the obtained T2 images, two regions-of-interest (ROI) were defined bilaterally, distinguishing the lesioned ventral striatum and the normal-appearing dorsal striatum (compare Additional file 2: Fig. S1). The within-ROI means of the analyzed parameters—T2IEW, MWF, FA and RD—were extracted across all five time points (Additional file 2: Fig. S1). At the end of the last MRI session (21 dpc), the rat was transcardially perfused under anesthesia and brain tissue was harvested for histology.

## Results

### Experimental ODS lesions in rats are widely remyelinated 3 weeks after lesion induction

**Fig. 1:**
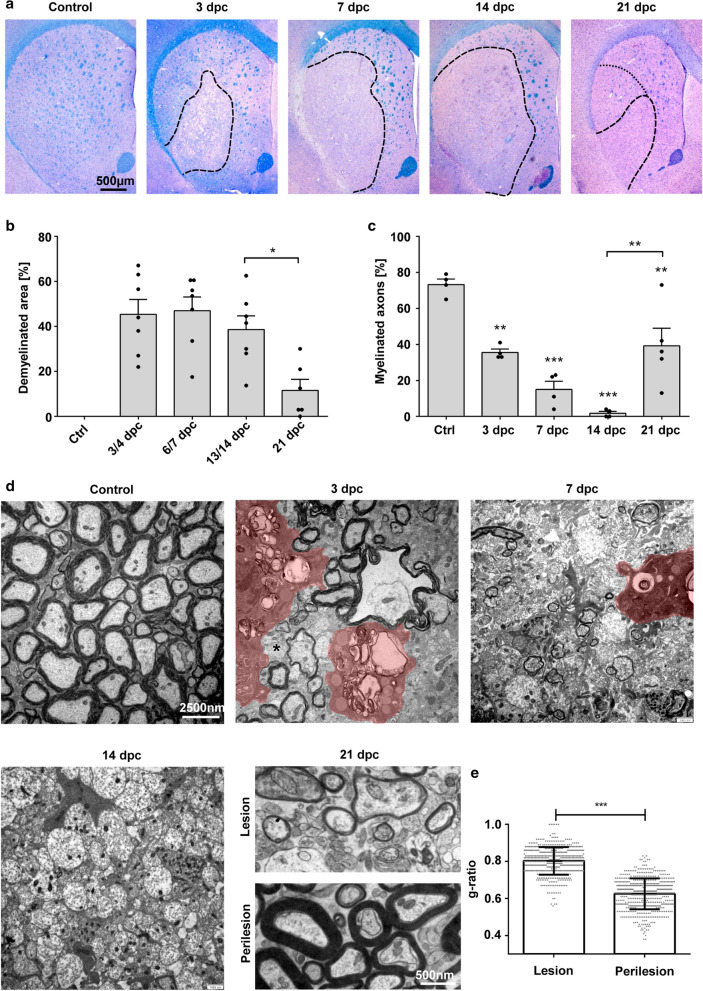
Evolution of experimental osmotic demyelinated lesions in the corpus striatum. **a** Representative micrographs of LFB/PAS-stained ODS lesions at different time points after the correction of serum sodium levels. The dashed line marks the lesion border, the dotted line indicates an already remyelinated area, indicated by pale LFB-staining, at 21 dpc; magnification × 20, scale bar 500 µm. **b** Demyelinated area as percentage of whole striatum (one dot represents one animal, mean ± SEM, one-way ANOVA and Tukey’s multiple comparison, *p < 0.05). **c** Percentage of myelinated axons per viewing field at different time points. Fiber tracts in the lesion center were analyzed (one dot represents one animal, mean ± SEM, one-way ANOVA and Tukey’s multiple comparison, p < 0.0001; **p < 0.01, ***p < 0.001). **d** Representative electron micrographs of transverse sections of striatal fiber tracts showing intralesional fibers at 3, 7, 14, 21 dpc, as well as perilesion fibers at 21 dpc and control animals. magnification × 3150. **e** g-ratios at 21dpc lesion versus perilesion (n = 5, one dot represents one axon, mean ± SD, t-test, p < 0.0001***)

To study the time course and cellular mechanisms of myelin regeneration in osmotic demyelination, we induced astrocyte and subsequent oligodendrocyte loss and demyelination by the rapid correction of severe hyponatremia in rats [11, 98]. Demyelinated lesions were present in the corpus striatum of all animals, as well as the claustrum, external capsule, neocortex and anterior commissure in a subset of animals as summarized in Table 3. In the corpus striatum, demyelinated lesions were clearly visible upon evaluation in LFB-PAS staining at 3, 7, 14 and 21 days post-correction (dpc) (Fig. 1a). A significant reduction in the size of the demyelinated lesion could be observed between days 14 and 21 post-correction (p = 0.0182) (Fig. 1b). Ultrastructural examination of the striatal fiber tracts showed a significantly reduced number of myelinated axons inside the lesion, compared to naïve controls (Fig. 1c). At 3 and 7 dpc, remaining myelin sheaths appeared vacuolized with microglial cells carrying myelin debris, while at 14 dpc barely any myelinated axons were present. At 21 dpc, thinly myelinated axons could be found scattered throughout the lesion, whereas the perilesion area presented regularly myelinated fiber tracts comparable to controls (Fig. 1d). At 21 dpc, g-ratios from axons inside the lesion were significantly increased when compared to perilesional axons, confirming regenerated myelin sheaths (lesion: 0.80 ± 0.08, perilesion: 0.63 ± 0.08 cells/mm^2^; p < 0.0001) (Fig. 1e). Increased g-ratios were independent from axon diameters (data not shown).

**Table 3:** Anatomic location of demyelinated lesions in ODS rats

Days post-correction	Corpus striatum	Claustrum	External capsule	Neocortex	Anterior commissure
3–4	7/7	6/7	2/7	0/7	2/7
6–7	7/7	7/7	6/7	2/7	0/7
13–14	7/7	5/7	2/7	0/7	0/7
21	4/6	3/6	0/6	0/6	2/6

Consistent with the histological findings, repeated magnetic resonance imaging (MRI) visualized the development of bilateral lesions in the ventral striatum, which manifested as increased signal intensity on T2-weighted images onwards from 7 dpc. Following sodium correction, the myelin water fraction (MWF) decreased within the lesion area, reached a minimum at 7 dpc and stabilized thereafter. Radial diffusivity (RD) followed a similar—but inverted—time course, with maximum values at 7 dpc and subsequent stabilization. The 7 dpc time point was also characterized by a marked decrease in fractional anisotropy (FA) and a prolongation of T2 relaxation times for myelin water and intra-/extracellular water (T2IEW), altogether indicating rapid lesion evolution until day 7 and ensuing tissue repair (Additional file 2: Fig. S1).

### Early loss of mature oligodendrocytes precedes myelin degeneration

**Fig. 2:**
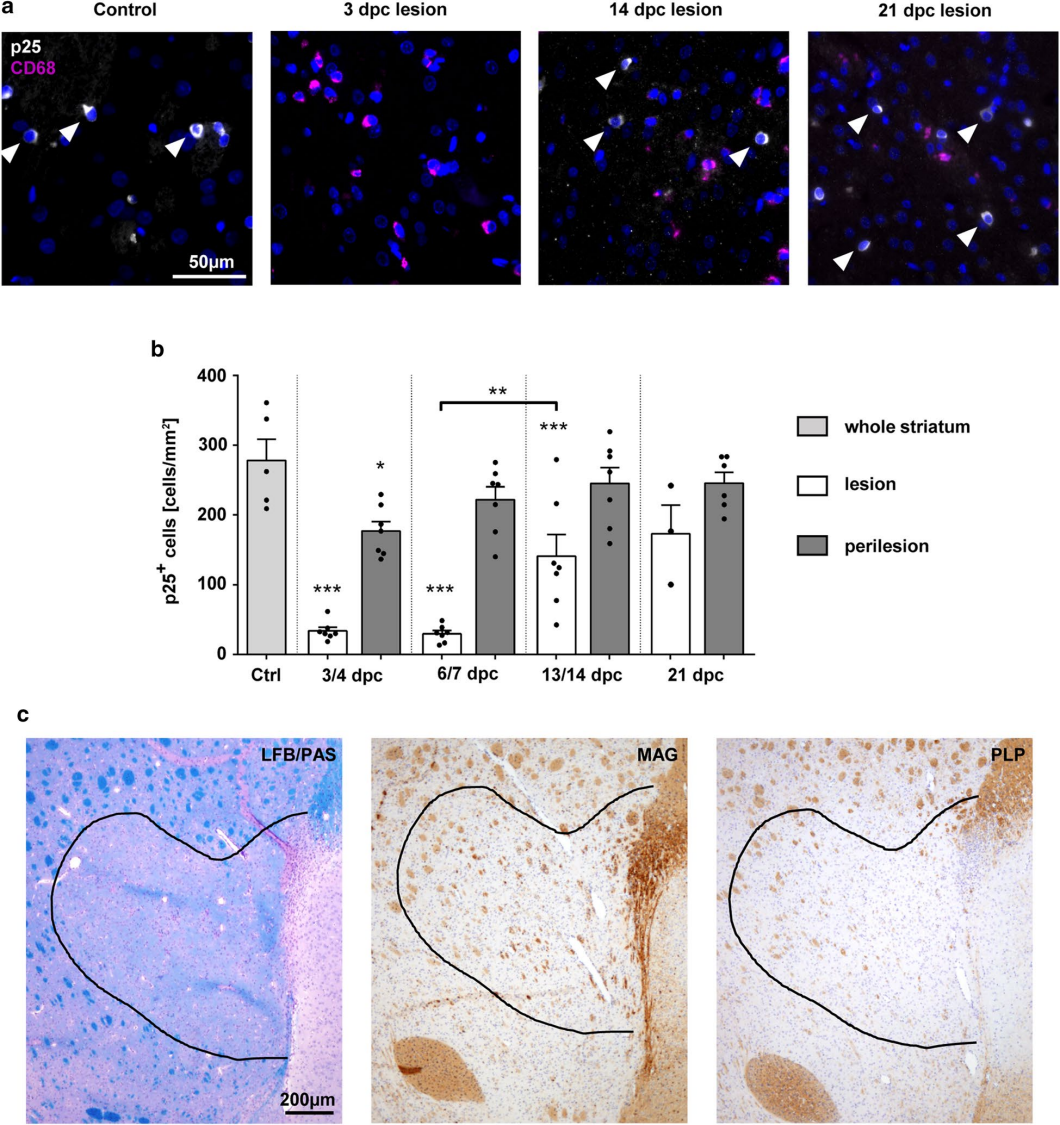
Mature oligodendrocytes reappear 2 weeks, myelin 3 weeks after lesion initiation. **a** Mature oligodendrocytes (TPPP/p25; white; arrowheads) and CD68+ activated microglia/macrophages (purple) at different time points after lesion induction. Nuclei are stained in blue (DAPI); original magnification × 200, scale bar 50 µm. **b** Quantification of TPPP/p25+ mature oligodendrocyte densities at different time points after correction of serum sodium levels (white bars = lesion, grey bars = perilesion, light grey bar = controls; one dot represents one animal, mean ± SEM, one-way ANOVA and Tukey’s multiple comparison, *p < 0.05, **p < 0.01, ***p < 0.001). **c** Representative ODS lesion at 21 dpc. LFB/PAS staining as well as anti-MAG and anti-PLP immunohistochemistry are shown. Dashed line marks the lesion border. Magnification × 40, scale bar 200 µm

To determine the time course of myelin-forming oligodendrocyte death and myelin degeneration, intra- and perilesional numbers of mature oligodendrocytes were quantified. The histological markers that allow the classification of oligodendroglial cells into different maturation states are listed in Table 4. We used CD68-positive activated microglia/macrophages to define lesion areas. Three out of 6 animals showed no sharply demarcated lesion at 21 dpc and were therefore excluded from the evaluation of intralesional cell densities. Inside the lesion, densities of TPPP/p25-positive mature oligodendrocytes were significantly reduced at 3, 7 and 14 dpc. At 21 dpc, lesional densities were comparable to healthy controls. Between 7 and 14 dpc a significant increase in intralesional densities of TPPP/p25-positive oligodendrocytes from 29.8 ± 12.2 to 140.9 ± 81.1 cells/mm^2^ was observed (p = 0.0047). Perilesional densities were slightly decreased at 3 dpc but reflected control levels at all other investigated time points (Fig. 2a, b). The repopulation of the demyelinated lesion with mature oligodendrocytes was accompanied by a sequential return of myelin proteins. After 3 weeks of recovery, lesions appeared still largely negative for LFB, but already positive for MAG and slightly positive for PLP (Fig. 2c), thus presenting typical histological hallmarks of recent remyelination [94].

**Table 4:** Oligodendroglial differentiation and corresponding markers used in this study

Cell type	Markers
Neural progenitor cells (oligodendrocyte progenitors)	Olig2, Nestin (PDGFRα)
Oligodendrocyte precursor cells	Olig2, PDGFRα, NG2
Immature/pre-myelinating oligodendrocytes	Olig2, BCAS1
Actively myelinating oligodendrocytes	Olig2, BCAS1, MAG
Mature oligodendrocytes	Olig2, TPPP/p25

### Early lesions are rapidly repopulated by NG2-positive OPCs

**Fig. 3:**
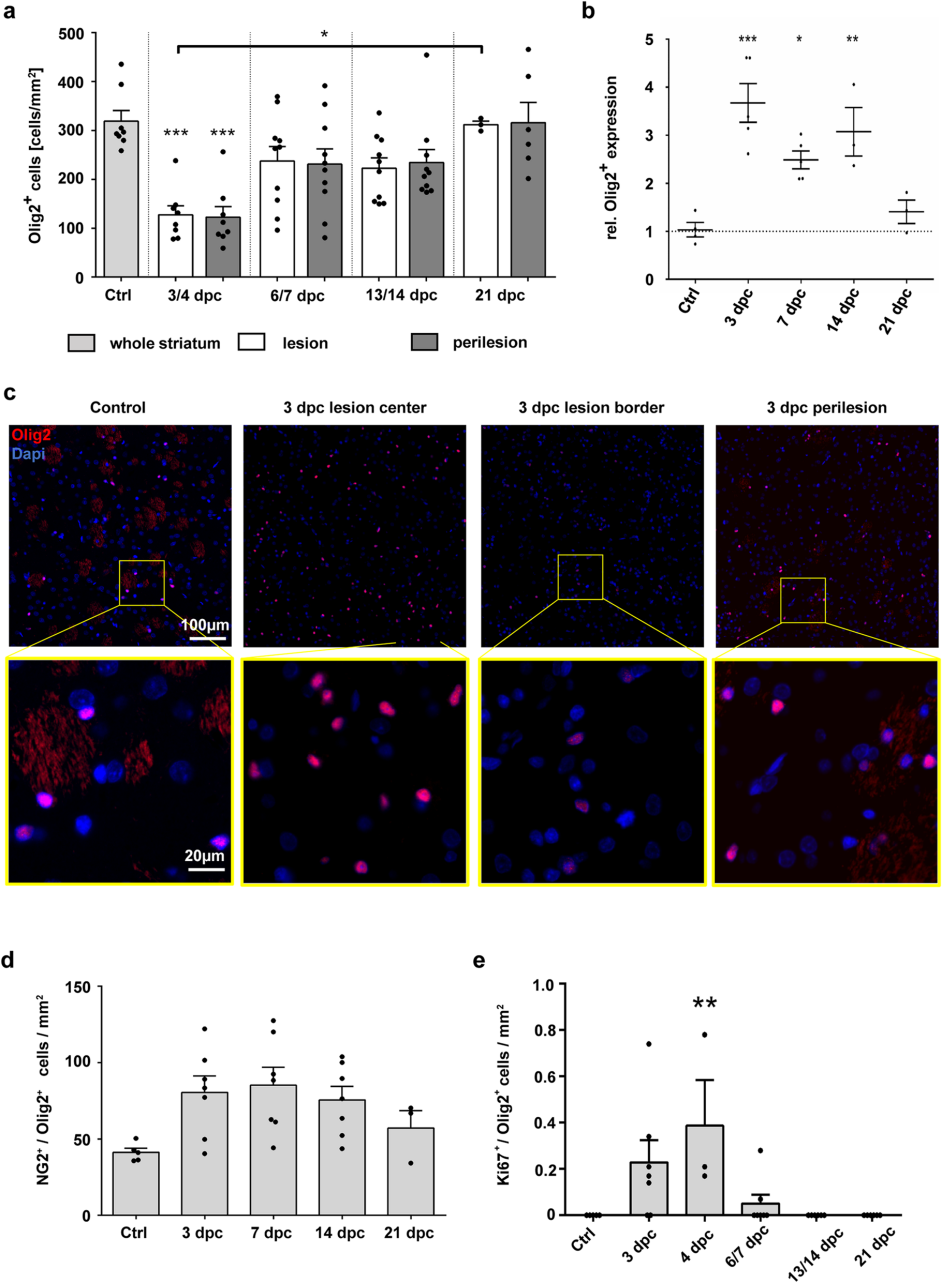
Olig2+ oligodendroglia in experimental ODS lesions. **a** Quantification of Olig2+ cells at different time points in lesion (white) and perilesion (grey) areas, as well as controls (light grey). **b** Relative Olig2 mRNA expression in the lesion center at 3, 7, 14 and 21 dpc compared to controls. Expression levels are normalized to the mean number of Olig2+ cells at each time point. **c** Representative pictures of Olig2+ cells (red); nuclei are stained in blue (DAPI). Upper row original magnification × 200, scale bar 100 µm, lower row shows magnifications of the framed areas above, scale bar 20 µm. **d** Density of NG2/Olig2 double-positive OPCs in ODS lesions. **e** Quantification of Olig2/Ki67 double-positive proliferating oligodendrocytes in the entire striatum at different time points. If not stated otherwise, one dot = one animal; one-way ANOVA and Tukey’s multiple comparison; mean ± SEM (*p < 0.05, **p < 0.01, ***p < 0.001)

Next, we aimed at investigating the cellular processes underlying lesion repair and asked whether OPC densities were affected by osmotic tissue damage. Immunohistological investigation revealed that densities of cells positive for the pan-oligodendroglial marker Olig2 were significantly reduced at 3 dpc in lesion as well as perilesion areas. Cellular densities were slightly reduced at 7 and 14 dpc, not reaching statistical significance, returning to control level at 21 dpc (Fig. 3a). Semiquantitative RT-PCR on RNA isolated from the lesion center revealed that levels of Olig2 mRNA expression were significantly increased at 3, 7 and 14 dpc and returned to control levels at 21 dpc (Fig. 3b). High expression levels of Olig2 indicate an early maturation phase of lesional oligodendrocyte populations and have been linked to reactive OPCs in the demyelinated adult CNS [25]. At 3 dpc, oligodendroglial cells in the lesion center showed an intense Olig2 immunoreactivity, whereas oligodendrocytes located at the lesion border had a low Olig2 immunoreactivity (Fig. 3c). This indicated an early OPC reaction that was mainly located in the lesion center, while the lesion border was still mainly populated by mature oligodendrocytes [47]. In the perilesion area and in controls, a mixture of cells with high and low Olig2 immunoreactivity was detectable (Fig. 3c). We observed that densities of Olig2/NG2 double-positive OPCs were significantly increased in the lesion at 3 and 7 dpc, and markedly (but not significantly) increased at 14 dpc (p = 0.086) when compared to controls (ctrl: 41.2 ± 5.9, 3/4dpc: 80.5 ± 10.7, 6/7dpc: 85.2 ± 11.8, 13/14dpc: 75.6 ± 8.9 cells/mm^2^; Fig. 3d). Densities decreased again to control levels at 21dpc, when lesion repair seemed to be largely completed (Fig. 3d). It has been previously demonstrated that upon demyelination, OPCs enter an activated state, allowing them to proliferate, migrate and differentiate (reviewed in [91]). We verified the early activation of intralesional OPCs by immunolabelling for proliferating cell nuclear antigen (PCNA; Ki67). Quantification of Ki67/Olig2 double-positive cells revealed proliferating oligodendrocytes inside the corpus striatum parenchyma at 3/4 dpc and in 2 out of 7 animals at 6/7 dpc (Fig. 3e).

### BrdU injection traces parenchymal OPCs that proliferated in early astrocyte-depleted lesions

**Fig. 4:**
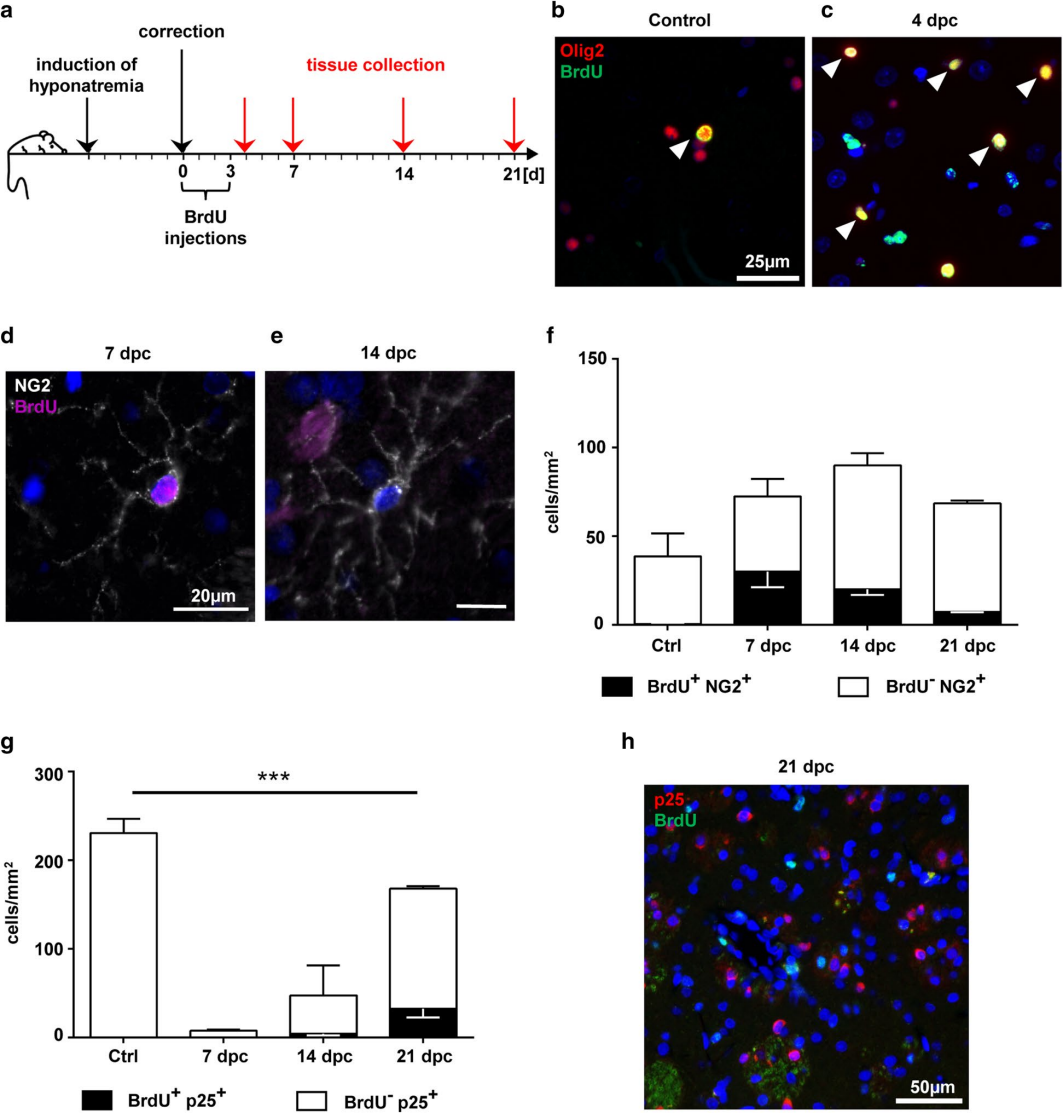
BrdU-labelling identifies early proliferating oligodendrocyte lineage cells. **a** Experimental setup of BrdU injections from 12 h until 3 d post-correction. Tissue was harvested at 4, 7, 14 and 21 dpc, as indicated by red arrows. **b**, **c** Immunofluorescence double labeling of BrdU/Olig2 double-positive cells (arrowheads) in a control animal (**b**) and the striatal ODS lesion area at 4 dpc (**c**). BrdU = green, Olig2 = red, DAPI = blue, magnification × 400, scale bar 50 µm. **d-e** NG2/BrdU immunofluorescence double labeling in the lesion 7 (**d**) or 14 dpc (**e**). BrdU = purple, NG2 = white, DAPI = blue, magnification × 400, scale bar 20 µm. **f**, **g** Quantification of cellular densities of NG2 + OPCs (**f**) and mature TPPP/p25+ oligodendrocytes with and without BrdU immunoreactivity at different time points inside the lesion. Black bars = BrdU+ cells, white bars = BrdU^−^ cells (mean ± SEM, two-way ANOVA and Tukey’s multiple comparison, ***p < 0.001). **h** Mature TPPP/p25+ oligodendrocytes are widely BrdU-negative at 21 dpc (TPPP/p25 = red, BrdU = green, magnification × 200, scale bar 50 µm)

To follow-up on oligodendrocytes that proliferated in the early (astrocyte-depleted) lesion stage, we performed repeated BrdU injections between 12 h and 3 days after the osmotic insult (compare Fig. 4a). Olig2-positive oligodendrocytes with incorporated BrdU could only sparsely be detected in control animals (Fig. 4b), whereas they were numerous within the lesion center at 4 dpc (Fig. 4c), thereby confirming early oligodendrocyte proliferation upon lesion initiation. To assess the maturation of these oligodendroglial cells, immunofluorescence multi-labeling with typical markers for different maturation stages was performed. At 7 dpc, BrdU/NG2 double-positive OPCs were present within the lesion, often showing a bipolar morphology (Fig. 4d), indicating recent proliferation and probably also migration [99]. At later time points, BrdU-negative/NG2-positive OPCs with the typical ramified morphology of OPCs were more abundant, indicating a replenishment of the parenchymal stem cell pool (Fig. 4e). At 7 dpc, nearly half of the intralesional OPCs showed BrdU incorporation. Numbers of BrdU-positive OPCs were gradually decreasing at later lesion stages, indicating further maturation of those cells (Fig. 4f). Although densities of TPPP/p25 positive mature oligodendrocytes nearly returned to control levels after 3 weeks, BrdU/p25 double-positive oligodendrocytes were infrequent (Fig. 4g, h), indicating that most early proliferating NG2-positive OPCs did not undergo final maturation.

### BCAS1-positive oligodendrocytes remyelinate experimental ODS lesions, but are mainly BrdU negative

**Fig. 5:**
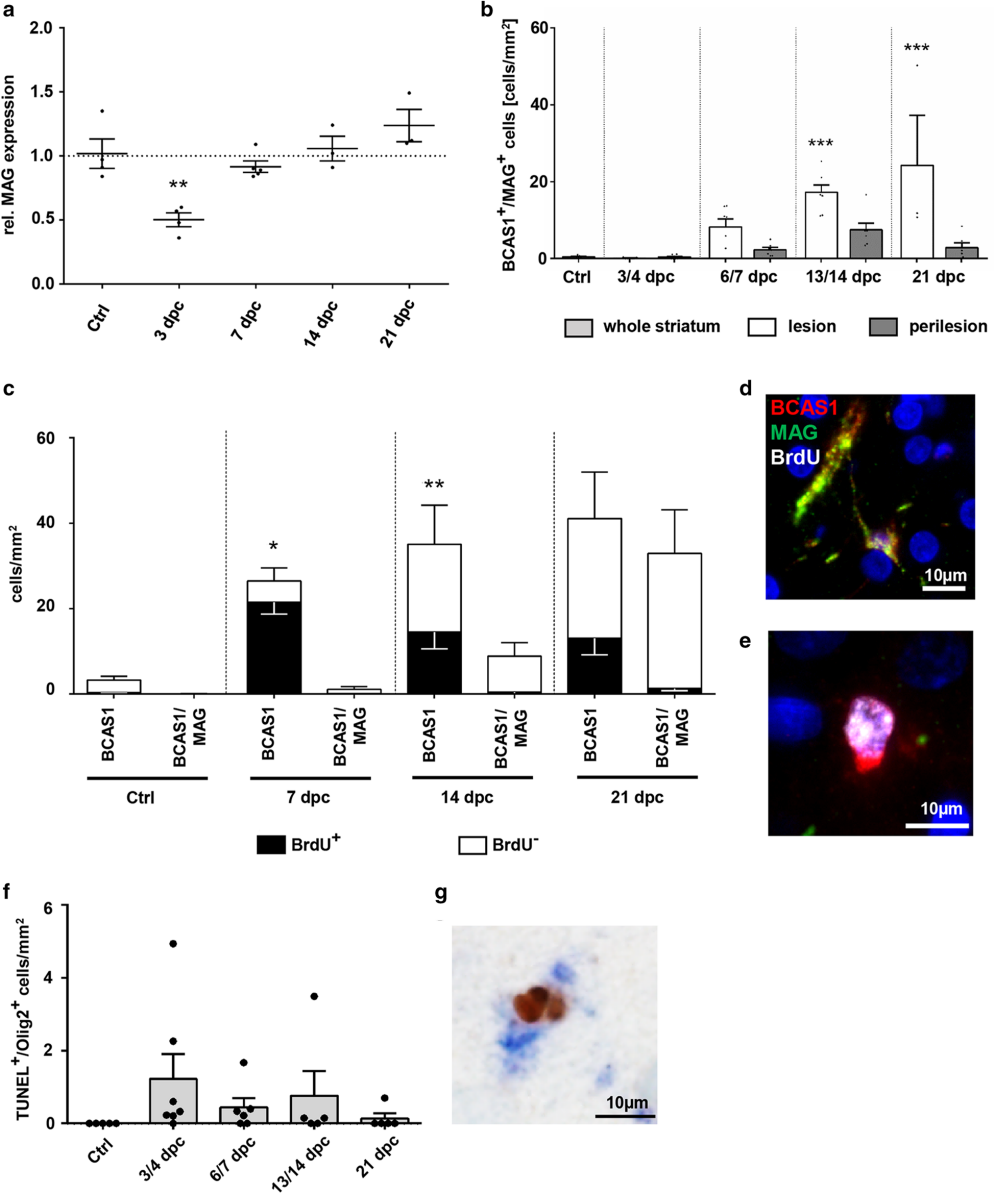
BCAS1-positive oligodendrocytes contribute to remyelination of ODS lesions. **a** Relative MAG mRNA expression in the lesion center at different time points compared to controls. Expression levels are normalized to the mean number of Olig2+ cells at this time point. **b** Quantification of BCAS1 +/MAG+ actively myelinating oligodendrocytes at different time points after lesion initiation. White bars = lesion, grey bars = perilesion, light grey bar = controls. **c** Quantification of BCAS1+ (left bar) and BCAS1+/MAG+ (right bar) oligodendrocytes with and without BrdU immunoreactivity at different time points inside the lesion. Black bars = BrdU+ cells, white bars = BrdU^−^ cells. **d**, **e** BCAS1/MAG/BrdU immunofluorescence triple labeling in an ODS lesion at 14 dpc. Depicted are an actively myelinating BCAS1+/MAG+ oligodendrocyte without BrdU labeling (**d**) and a BCAS1 +/MAG^−^ oligodendrocyte with nuclear BrdU labeling and dysfunctional morphology (**e**) (BrdU = white, BCAS1 = red, MAG = green, DAPI = blue, magnification × 400, scale bar 10 µm). **f** Olig2/TUNEL double-positive cells, indicating apoptotic oligodendrocytes. **g** Light microscopic BCAS1/BrdU double labeling depicting a double positive cell with typical apoptotic nuclear morphology. BCAS1 = blue, BrdU = brown, magnification × 400, scale bar 10 µm. If not stated otherwise, one dot = one animal; one-way ANOVA and Tukey’s multiple comparison; mean ± SEM (*p < 0.05, **p < 0.01, ***p < 0.001)

The proper differentiation of OPCs into myelinating oligodendrocytes has been identified as a limiting factor for efficient remyelination in MS [16, 58]. To further characterize the myelin protein expression expected to accompany remyelination, quantitative RT-PCR of mRNA encoding for myelin-associated glycoprotein (MAG), required for early myelinating oligodendrocyte-axon contact, was performed. At 3 dpc, normalized MAG mRNA expression levels were reduced by half compared to controls, substantiating that most oligodendrocytes were in a very early maturation state and not yet myelinating. At 7/14 dpc mRNA levels returned to, and slightly surpassed, control levels at 21 dpc (Fig. 5a), indicating ongoing remyelination at later lesion stages. Breast carcinoma-amplified sequence 1 (BCAS1) has recently been characterized as a marker protein for a pre-myelinating and myelinating oligodendrocyte subpopulation derived from NG2-positive OPCs [26]. Since the early loss and later upregulation of MAG-expression indicated active myelination, we performed a BCAS1/MAG double-labeling to detect actively myelinating oligodendrocytes. Actively myelinating oligodendrocytes were largely absent in controls as well as during early destructive lesion stages (3/4 dpc). At 6/7 dpc some BCAS1/MAG double-positive oligodendrocytes were detectable. Afterwards, densities increased further, leading to significantly increased cell numbers at 13/14 and 21 dpc (ctrl: 0.5 ± 0.4, 13/14dpc: 17.2 ± 5.1, 21dpc: 24.3 ± 22.5 cells/mm^2^; Fig. 5b), thus demonstrating remyelination of ODS lesions by BCAS1-positive oligodendrocytes. At all time points, active (re-) myelination was mainly observable at the lesion border.

Utilizing the BrdU injections described above, we then determined the contribution of OPCs that proliferated in early astrocyte-depleted lesion stages to remyelination. For this, we distinguished pre-myelinating BCAS1-positive/MAG-negative and actively myelinating BCAS1/MAG double-positive cells. At 7 dpc, when astrocytes were still nearly absent in the lesion (compare Additional file 3: Fig. S2a), overall densities of BCAS1-positive cells (per definition including pre-myelinating and actively myelinating cells) were already significantly increased compared to controls (control: 3.4 ± 0.8, 7 dpc: 53.9 ± 13.2 cells/mm^2^; p = 0.0007). Most of these cells showed the incorporation of BrdU and were distributed equally throughout the lesion, whereas actively myelinating oligodendrocytes were not yet detectable (Fig. 5c). This indicated that early proliferating, NG2-positive OPCs were able to differentiate into pre-myelinating oligodendrocytes within 1 week. It is known that BCAS1 positive oligodendrocytes present a characteristic star-shaped morphology with fine processes or a ‘t-shaped’ appearance due to the remyelination of a nearby axon (compare Fig. 5d, [26]). However, at 7 dpc, pre-myelinating BrdU/BCAS1 double-positive (MAG negative) cells in the lesion often presented a ‘dysfunctional’ morphology with few if any short ramifications (Fig. 5e).

At 14 as well as 21 dpc, actively myelinating BACS1/MAG double-positive oligodendrocytes were present. However, BrdU incorporation was rare among these cells (Fig. 5c). Though BrdU/BCAS1 double-positive cells were still numerous, they located preferably within the lesion center rather than at the lesion border. Such an accumulation of undifferentiated OPCs has also been reported for MS lesions [47]. The preferred presence of BCAS1/MAG double-positive cells at the lesion border indicates a myelin regenerative response directed from the rim towards the lesion center that was largely independent of cells derived from early proliferation in the intralesional parenchymal OPC pool. Moreover, using a TUNEL assay, we detected dying oligodendroglial lineage cells in the striatal tissue of ODS rats. TUNEL/Olig2 double-positive cells were present during early destructive lesion stages, but also throughout lesion repair (Fig. 5f). Histological investigation revealed non-ramified BCAS1/BrdU double-positive cells with condensed and fragmented nuclei, indicating apoptosis of dysfunctional OPCs (Fig. 5g).

### Onset of subventricular zone-dependent regeneration follows the parenchymal OPC response

Our data so far indicated that oligodendrocytes that remyelinate the lesion are mostly derived from a cellular source other than early proliferating parenchymal OPCs. As shown above, large demyelinated lesions with negligible numbers of mature oligodendrocytes were observed at 3 and 7 dpc. We analyzed the distribution of TPPP/p25-positive oligodendrocytes across the striatum, including lesion and perilesion areas, and performed spatial statistical analysis. The resulting repopulation pattern pointed at lesion repair from the subventricular zone (SVZ) towards the lesion center rather than a concurrent oligodendrocyte maturation throughout the whole lesion (Additional file 4: Fig. S3).

In general, 3 weeks after lesion induction, remyelinated axons could be found in the striatal grey and white matter. Although, it should be noted that especially the grey matter axons in the lesion center were not efficiently remyelinated at 21dpc, whereas grey as well as white matter regions near the lesion border showed quite efficient remyelination (Additional file 5: Fig. S4). The lesion densities of mature oligodendrocytes at 21dpc were, however, close to control levels throughout the lesion. This further underscores that remyelination was barely performed by locally proliferated, BrdU-positive oligodendrocytes that were already equally distributed throughout the lesion at very early time points, and mainly located in the lesion center.

Myelin regeneration by SVZ-derived myelinating oligodendrocytes has been demonstrated after cuprizone-induced demyelination, and was described as even more efficient than regeneration by parenchymal OPCs [104]. Therefore, we hypothesized that lesion repair in the striatal osmotic demyelinated lesions studied here could be carried out by SVZ-derived glial cells.

**Fig. 6:**
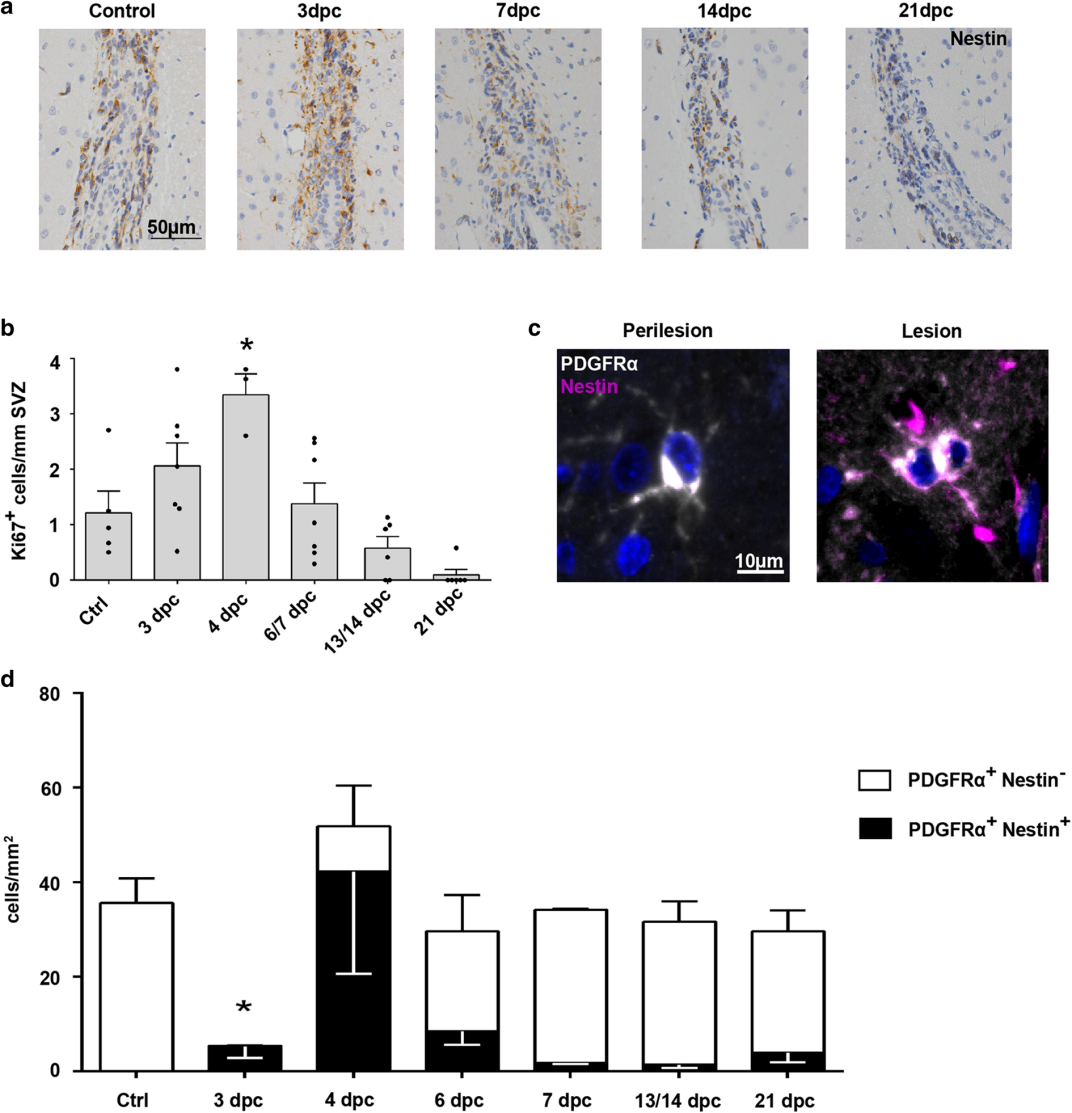
SVZ-derived lesion repair of striatal ODS lesions. **a** Representative microphotographs of the SVZ stained with anti-nestin antibodies at different time points (nestin = brown; nuclei = blue); Original magnification × 400, scale bar represents 50 µm. **b** Quantification of Ki67+ proliferating cells in the SVZ at different time points after ODS lesion induction. Immunopositive cells per mm SVZ length are plotted (one dot represents one animal, mean ± SEM, one-way ANOVA and Tukey’s multiple comparison, *p < 0.05). **c** Immunofluorescence double labeling with anti-PDGFRα and anti-nestin antibodies of perilesional and lesional OPCs at 7 dpc (PDGFRα = white, nestin = purple, DAPI = blue, magnification × 400, scale bar represents 10 µm). **d** Quantification of PDGFRα+ cells with and without nestin immunoreactivity at different time points inside the lesion. Black bars = nestin+ cells, white bars = nestin^−^ cells (mean ± SEM, two-way ANOVA and Tukey’s multiple comparison, *p < 0.05)

Following tissue damage, the type VI intermediate filament protein nestin is expressed in proliferation-capable neural progenitor cells in the SVZ [46]. Besides neurons, these cells can differentiate into glial cells, including OPCs, that are characterized by the expression of PDGFRα and nestin [62]. Immunohistochemistry confirmed nestin-positive cells in the SVZ of all investigated animals. At 3 dpc, nestin-positive cells were most abundant in the SVZ, decreasing below control levels, with very few nestin-positive cells at 21 dpc (Fig. 6a). Ki67-positive IHC indicated proliferating cells inside the SVZ. A basal proliferation was detectable in control animals; increased levels were seen at very early time points, especially at 4 dpc, decreasing afterwards with barely any proliferating activity at 21 dpc (Fig. 6b). In the early demyelinated lesion, PDGFRα-positive OPCs throughout the lesion showed co-expression of nestin, whereas perilesional OPCs were widely negative for nestin (Fig. 6c). Quantification revealed significantly decreased densities of overall PDGFRα-positive OPCs in the lesion at 3 dpc (control: 35.6 ± 5.2, 3 dpc: 5.4 ± 2.5 cells/mm^2^; p = 0.0135). At 4 dpc, OPC densities had greatly increased compared to the previous day (4 dpc: 51.8 ± 19.3 cells/mm^2^), and still ~ 80% of immature oligodendrocytes expressed nestin. Although GFAP-positive astrocytes were nearly absent in early lesions and did not reappear until 7 dpc (Additional file 3: Fig. S2a), few small and faintly GFAP-positive cells with mono- or bipolar morphology could be found at 3/4 dpc. These cells were often weakly Olig2-positive (Additional file 3: Fig. S2b), possibly indicating a very early maturation state of SVZ-derived neural stem cells towards glial differentiation. At later timepoints, OPC densities returned to control levels with few nestin-positive cells mainly located at the lesion border, which were completely absent in control animals (Fig. 6d).

The proliferation in the SVZ mainly occurred at 3/4 dpc when BrdU injections were finished, leading to an oligodendroglial cell population that was mostly negative for BrdU. Accordingly, we observed that PDGFRα/nestin double-positive OPCs were widely BrdU-negative (data not shown) and therefore concluded that the SVZ-derived response was induced after the parenchymal response. Although additional cellular sources of actively myelinating oligodendrocytes (e.g. transdifferentiated astrocytes) cannot be excluded, a substantial contribution of SVZ-derived cells to lesion regeneration can thus be assumed, especially as lesion repair was shown to be oriented from the SVZ towards the lesion center. In line with the appearance of SVZ-derived glial cells, the activated (but widely dysfunctional) parenchyma-derived oligodendrocyte population was most likely no longer needed in the lesion. Therefore, those cells could either undergo apoptosis or return to their quiescent state, as indicated by a regular density of ramified NG2-positive OPCs in late-stage lesions (compare Fig. 4e).

### Human central pontine myelinolysis (CPM) lesions

**Fig. 7:**
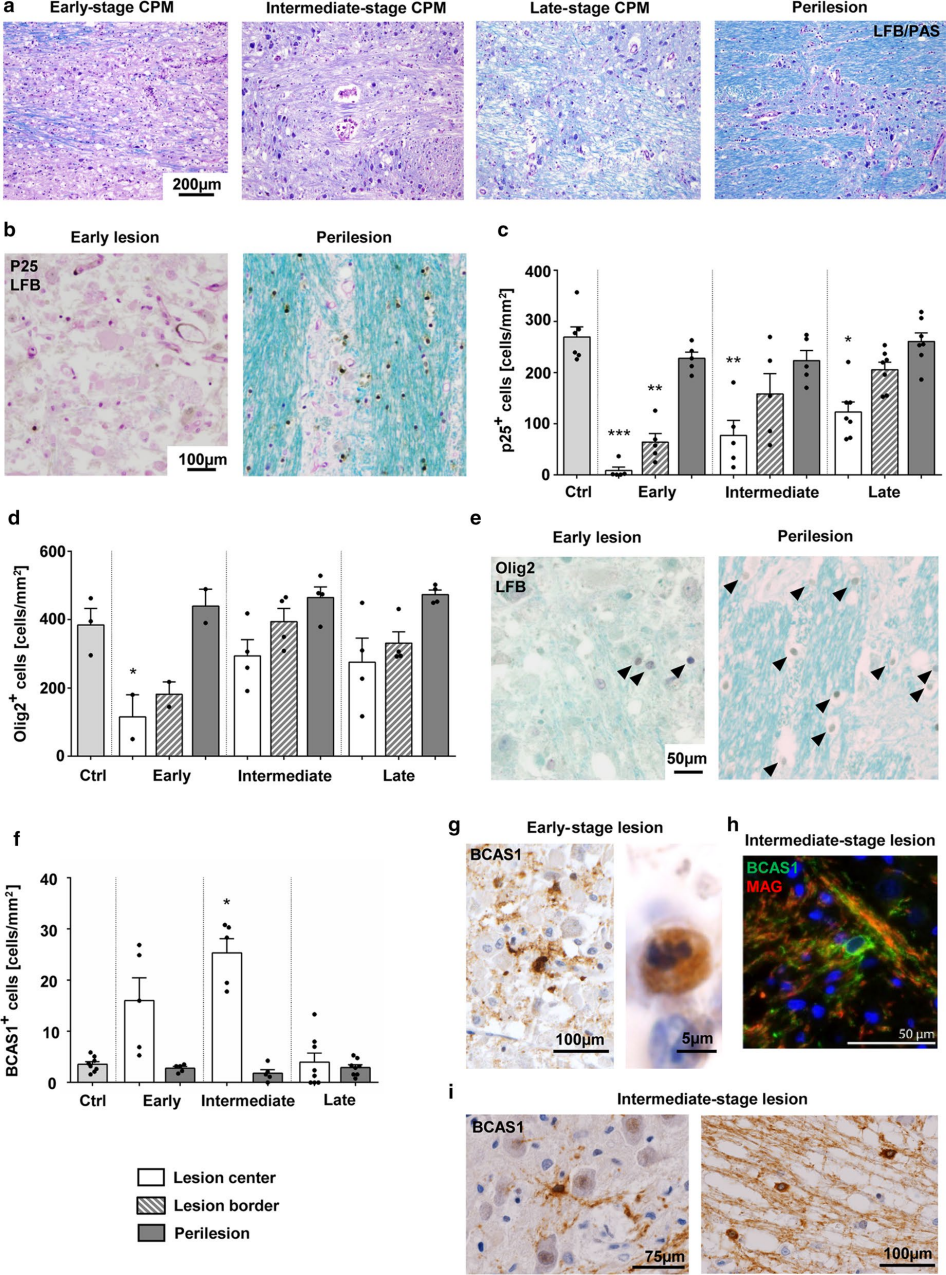
De- and remyelination in human CPM lesions**. a** Representative micrographs of human pontine CPM lesions stained with LFB/PAS. Typical early-, intermediate- and late-stage lesions are shown. The far right picture shows the perilesion area of the late-stage lesion depicted. Magnification × 100, scale bar represents 200 µm. **b** LFB staining in combination with immunohistochemistry for mature oligodendrocytes (TPPP/p25). Lesion and perilesion from the same early CPM case are shown. Magnification × 200, scale bar represents 100 µm. **c**, **d** Quantification of TPPP/p25+ (**c**) and Olig2+ (**d**) oligodendrocytes in different areas of early, intermediate and late stage CPM lesions compared to controls. White bars = lesion, grey bars = perilesion, striped bars = lesion border. **e** LFB staining combined with Olig2 immunohistochemistry of an early stage CPM lesion and the adjacent perilesion area. Arrowheads indicate Olig2+ nuclei. Magnification × 400, scale bar represents 50 µm. **f** Quantification of BCAS1+ oligodendrocytes in different areas of early-, intermediate- and late-stage CPM lesions in comparison to controls. **g**, **i** Typical cellular morphology of BCAS1-immunostained oligodendrocytes in early- (**g**) and intermediate-stage lesions (**i**). A BCAS1+ cell with fragmented nuclei is depicted (**g**, right picture). Original magnification × 400. **h** BCAS1/MAG immunofluorescence double labeling in an intermediate stage human CPM lesion. BCAS1 = green, MAG = red, DAPI = blue; magnification × 400, scale bar represents 50 µm. c,d,f dots correspond to individual patients. Bars = mean ± SEM and statistical analysis with one-way ANOVA followed by Tukey’s post hoc test for multiple comparison; *p < 0.05, **p < 0.01, ***p < 0.001

Based on findings from our animal model and previously published reports on human CPM, we staged a cohort of human CPM lesions, investigating astrocytes, myelin and macrophages. Staging criteria are outlined in Additional file 1: Table S2. Of the 18 CPM cases with clearly visible lesions in the pons, 5 lesions matched the criteria for early-, 5 for intermediate- and 8 for late-stage lesions. In experimental osmotic demyelination, astrocytes were shown to be especially vulnerable to the rapid rise of sodium levels after correction of hyponatremia, leading to their preferential loss (reviewed in: [64]). In line with these data, early human CPM lesions showed a prominent reduction in the density of GFAP-positive astrocytes. Remaining GFAP-positive astrocytes within the lesions often had a bipolar morphology and were negative for the water channel AQP4, indicating that these cells had recently repopulated [72]. In intermediate-stage lesions, the density of GFAP-positive astrocytes returned to control levels or was even increased, with a proportion of cells still showing a bipolar morphology. In 3/5 intermediate-stage cases, the lesion center was AQP4 negative. Late lesions contained mainly star-shaped, AQP4-positive astrocytes with typical reactive morphology (Additional file 6: Fig. S5). In early lesions, the loss of astrocytes was accompanied by the influx of abundant foamy, KiM1P-positive macrophages/activated microglia, which also phagocytosed myelin (Additional file 6: Fig. S5). Remnants of LFB-positive myelin sheaths were still found to a variable degree (Fig. 7a). In intermediate-stage lesions the number of KiM1P-positive cells slightly decreased, and phagocytes had a less foamy morphology. LFB was completely absent from late-stage lesions or showed signs of remyelination (Fig. 7a). In late-stage lesions, KiM1P-positive cells were rather small and round or ramified. Pale LFB staining was often detected throughout the lesion, indicating remyelination (Fig. 7a).

In line with the results obtained in experimental ODS lesions, loss of mature oligodendrocytes was an early feature of human CPM lesions. Indeed, immunohistochemistry for TPPP/p25 showed a significant reduction in cell densities within lesions while densities in perilesion areas remained unaltered. Of note, densities gradually increased from 8.5 ± 15.9 cells/mm^2^ in early- to 123.0 ± 51.8 cells/mm^2^ in late-stage lesions (compared to 269.7 ± 48.9 cells/mm^2^ in controls) (Fig. 7b, c). Not only a reduction in mature oligodendrocytes, but also in oligodendroglia lineage cells in general was detected within lesions using Sox10 and Olig2 immunohistochemistry. Also here, densities within lesions did not reach control levels but showed an increase from early- to late-stage lesions (early: 115.2 ± 91.6 cells/mm^2^, late: 275.6 ± 139.8 cells/mm^2^, control: 384.0 ± 83.8 cells/mm^2^; Fig. 7d, e).

Already in early astrocyte-depleted CPM lesions, BCAS1-positive cells were detectable throughout the lesion (Fig. 7f), mainly in a pre-myelinating stage, as BCAS1/MAG double-positive cells were only found in one single early case at the lesion border. Comparable to early experimental ODS lesions, BCAS1-positive oligodendrocytes in early lesions often presented a dysfunctional morphology with few ramifications or even condensed and fragmented nuclei (Fig. 7g). Densities of BCAS1-positive cells were significantly increased in intermediate-stage lesions (Fig. 7f). In 4/5 intermediate-stage CPM lesions in which astrocytes widely repopulated the lesion, several actively myelinating cells double-labelled for BCAS1/MAG were observed (Fig. 7h). Here in general, BCAS1-positive oligodendrocytes presented the typical morphology of pre-myelinating or actively myelinating oligodendrocytes (Fig. 7i). No actively myelinating BCAS1-positive cells were observed in late-stage CPM, and densities of BCAS1-positive oligodendrocytes returned to control levels (Fig. 7f), indicating no further attempts at remyelination.

In human CPM cases, nestin-positive cells were neither detected in the pontine lesions nor in the wall of the fourth ventricle, whereas they were frequent in the wall of the fetal fourth ventricle (Additional file 7: Fig. S6), suggesting that nestin-positive cells may not substantially contribute to myelin regeneration in human pontine CPM lesions.

## Discussion

It was recently shown that the impaired differentiation of oligodendrocytes in MS lesions is likely due to extrinsic rather than cell-intrinsic factors [92]. Moreover, several studies demonstrated that the crosstalk between astrocytes and oligodendrocytes is indispensable for migration and maturation of OPCs during development. Consequently, the proper interplay with astrocytes is essential during replenishment with mature oligodendrocytes and remyelination in demyelinated lesions. In the lesion, the composition of signal molecules, secreted by other glial cells, determines whether OPCs undergo maturation to myelin-forming cells or remain as precursors (reviewed in: [23, 67]). Astrocytic secretion of the leukemia inhibitory factor (LIF) was determined as potent activator of myelination [39, 42]. Other astrocytic factors, e.g. PDGF, were also proven instrumental for OPC activation and maturation [7, 75], thus suggesting a tight regulation of oligodendroglial populations by astrocytes. Furthermore, connexins form gap junctions that enable direct astrocyte-oligodendrocyte communication by the free flow of small molecules, but they were found to be disrupted during lesion formation in CPM, but also in other neurodegenerative diseases, e.g. in NMO and MS lesions or Baló’s concentric sclerosis [30, 57]. As expected, upregulation of connexin 43 is observable in remyelinating MS lesions [57], further substantiating the role of gap junction-mediated astrocytic support for oligodendrocyte maturation. Although there is ample evidence of a crucial role of astrocytes during remyelination, there are only few studies on the cellular mechanisms of remyelination in demyelinated lesions with primary astrocyte damage or loss.

It is known that the remyelination capacity of NMO lesions that—like CPM—are characterized by a primary astrocytic insult, is limited [38, 100, 105]. One reason for this might be impaired oligodendroglial differentiation in the absence of functional astrocytes (see review [100]). This hypothesis has been substantiated by experiments on ex vivo brain cultures treated with recombinant antibodies from MS or NMO patient CSF in combination with human complement. It could be demonstrated that axons in demyelinated brain culture lesions treated with MS rAb were rapidly remyelinated, whereas lesions in NMO rAb-treated tissue were repopulated with astrocytes and pre-myelinating oligodendrocytes, but did not show substantial remyelination of preserved axons [52].

In our study, we investigated pontine lesions of 18 CPM patients which we stratified into early, intermediate and late disease stages by histopathological criteria. Studies in rodent models demonstrated the widespread absence of oligodendrocytes 24–48 h post-correction [11]. However, early human lesions contained Olig2-positive cells—albeit in significantly reduced densities—which suggests that lesion repair had already been initiated and oligodendrocytes had started to repopulate at this early lesion stage. Mature oligodendrocytes were nearly absent, indicating that Olig2-positive oligodendrocyte lineage cells must be OPCs or early differentiated oligodendrocytes. Compared to controls, pre-myelinating BCAS1-positive oligodendrocytes were increased. Although one early case showed few BCAS1/MAG double-positive cells, indicating active remyelination, most BCAS1-positive cells showed a dysfunctional morphology with few short ramifications or even condensed or fragmented nuclei. In intermediate-stage lesions where astrocyte repopulation was more advanced, BCAS1-positive cells were even more frequent and showed more ramifications, in part co-expressing MAG. Therefore, it can be concluded that lesion regeneration and in particular myelin repair is mainly carried out in this stage of lesion evolution. In contrast, late-stage lesions presented low densities of BCAS1-positive cells, increased numbers of mature oligodendrocytes and thin myelin, indicating that repair processes were largely completed.

Strikingly, we found that early CPM lesions harbored mainly dysfunctional pre-myelinating oligodendrocytes. Talbott and colleagues showed that OPCs failed to remyelinate astrocyte-free lesions in the rat spinal cord [95], underpinning the assumption that the lack of astrocytes in early CPM lesions might be the cause of the incomplete differentiation of pre-myelinating oligodendrocytes. A number of studies failed to demonstrate significant astrocytic loss in MS lesions [17, 71, 79]. Nevertheless, a recent study revealed astrocytic loss in a subset of early MS lesions as well as the repopulation of active lesions with AQP4-negative/GFAP-positive astrocytic precursors, similar to those detectable in our CPM lesions and in non-necrotic NMO lesions [74]. In addition, not only a lack of astrocytes but also functional impairment should be considered as contributing to inefficient remyelination of MS lesions.

To gain further understanding of lesion repair processes, we used an ODS rat model. In contrast to human CPM, demyelinated lesions in rats are mainly localized in the corpus striatum. This is thought to be due to differences in the anatomic architecture of the human versus rat brain [44]. Histopathologic examination points out a rapid repopulation of the astrocyte-free demyelinated lesion with OPCs, which reflects previous findings in experimental NMO [103]. Experiments revealed that most of the OPCs that repopulated the lesion were newly formed. Although proliferating oligodendrocytes are rare in the healthy adult rodent brain, a parenchyma-resident oligodendrocyte population marked by the expression of NG2 undergoes slow but constant proliferation [37, 50]. These cells, also referred to as adult or parenchymal OPCs, retain their ability to undergo maturation and were found to proliferate in demyelinating lesions that are successfully remyelinated afterwards [6, 41, 49, 60, 84]. It has been demonstrated that LPS-induced demyelinated lesions in the rat spinal cord are repaired by parenchymal, NG2-positive OPCs that undergo proliferation outside the lesion up to 48 h after lesion induction, and then migrate into the lesioned area where they continue to proliferate [99]. In line with these data, we detected proliferating oligodendrocyte lineage cells throughout lesion repair, but especially in the early lesion stages. Olig2-positive cells in the early lesion were often NG2/BrdU double-positive and showed a bipolar morphology, as expected for cells that had recently migrated into the lesion. However, although newly formed oligodendrocytes rapidly gained BCAS1 expression, they only partially entered into an actively myelinating state, or showed TPPP/p25 expression, which would indicate final maturation. Moreover, BCAS1/BrdU double-positive cells seemed to have halted their maturation, showing a dysfunctional morphology as also observed in early human CPM lesions. Apoptotic oligodendrocytes were present throughout lesion repair, suggesting a sorting out of immature oligodendrocytes in experimental ODS lesions, including dysfunctional BCAS1/BrdU double-positive cells.

Similar to human lesions, experimental ODS lesions also showed active remyelination, characterized by an increased density of BCAS1/MAG double-positive oligodendrocytes between 2 and 3 weeks after lesion induction (when astrocytes had already widely repopulated the lesion). By 3 weeks, lesion size had significantly decreased when compared to the maximal lesion expansion 1 week after lesion induction. Furthermore, densities of mature oligodendrocytes closely resembled control levels, and thin myelin was found histologically and ultrastructurally, implying that myelin regeneration was nearly completed. We further aimed at depicting lesion evolution and repair in the living animal and performed a follow-up MRI study over 21 days. Here we were able to observe tissue edema as a prolongation of the T2 relaxation time of about 15%, which was already visible on the T2-weighted images on 7 dpc, and was clearly apparent on the T2IEW map. The histologically confirmed demyelination was reflected by a reduction in the MWF. As shown before, MWF strongly correlates with histological myelin stains and is regarded as an in vivo myelin marker [55]. Similarly, the observed FA reduction and the RD increase pointed towards demyelination, as found in other studies in comparable animal models [10, 89]. All parameters showed a regression of the lesion already at 14 dpc, although to a different extent for the specific methods applied here. While FA and T2IEW almost returned to baseline, the parameters expected to be most specific for myelin, namely RD and MWF, showed only mild recovery, if at all. The histologically and ultrastructurally detectable thin myelin was interestingly not sufficient to be detectable by the myelin-related MR-parameters. Noteworthy, even the normal-appearing striatum showed consistently reduced values of MWF, which could be explained by a general increase in the intra- and extracellular water fractions. Further experiments would be needed to confirm whether this is a systematic effect of the intervention. In order to disentangle the contribution of the different pathologies including cell density, myelin content and extracellular water follow up studies may exploit multiparameter approaches [10, 63]. As the quite efficient lesion repair observed both histologically and by MRI contrasted with the inefficient maturation of parenchymal OPCs, we had to consider another cellular source of myelinating oligodendrocytes.

**Fig. 8:**
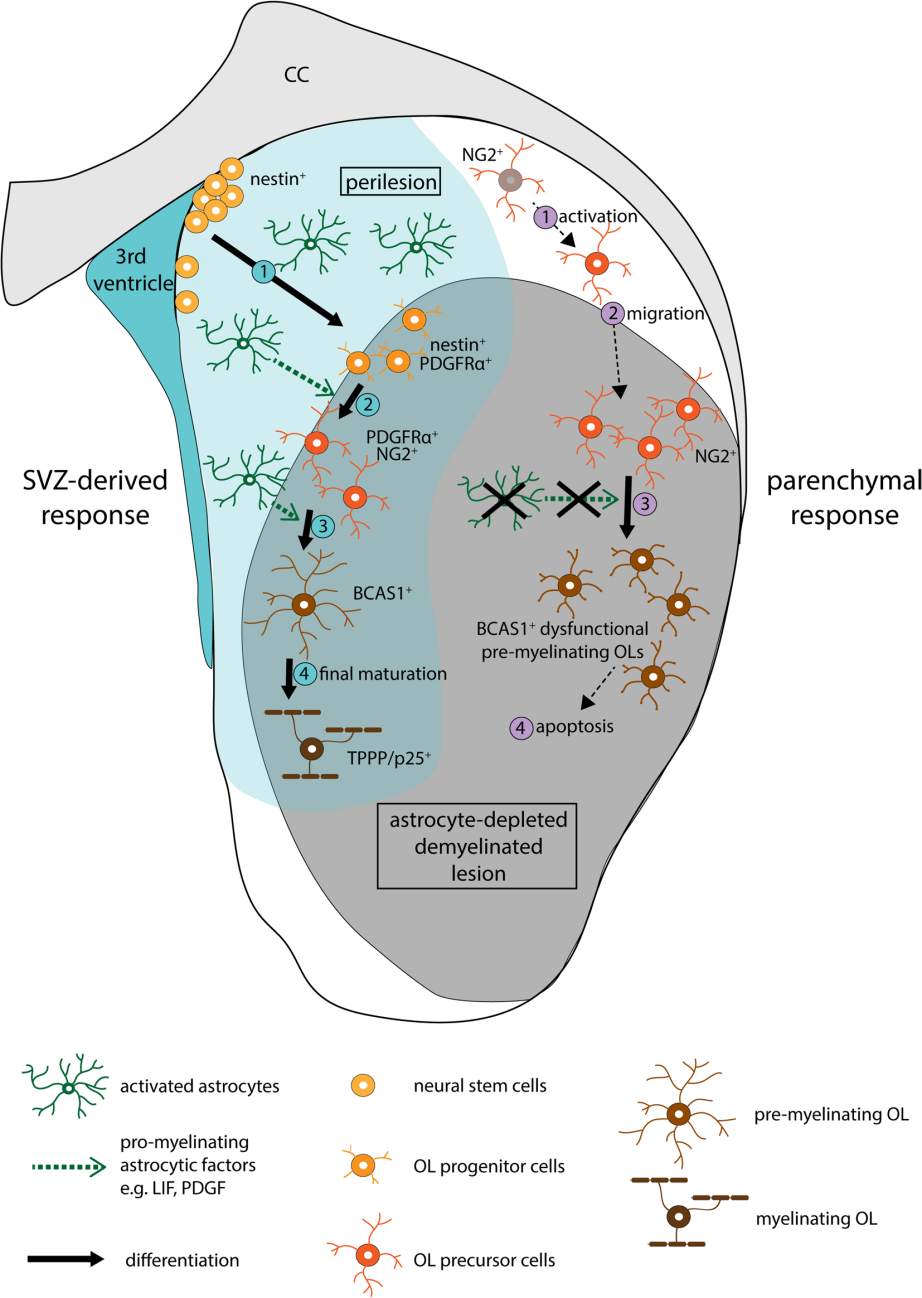
Graphical summary. The proposed dual mode of repair of astrocyte-depleted experimental ODS lesions in the striatum is shown. The initial parenchymal response is depicted on the right and marked by pink numbers. NG2-positive oligodendrocyte precursor cells (OPCs) are activated (1) and migrate into the lesion center (2), where they proliferate and repopulate the lesion. In the absence of functional astrocytes, OPCs further differentiate to BCAS1-positive pre-myelinating oligodendrocytes with a dysfunctional morphology (3). Most of those cells fail to undergo further maturation and enter apoptosis (4). After the initial repopulation of the lesion with OPCs, the SVZ-derived response (shown on the left in blue) takes place. SVZ-derived oligodendroglial progenitor cells are formed by neural stem cells located close to the third ventricle and migrate towards the lesion (1). At the lesion border, they differentiate into fully mature myelinating oligodendrocytes in the presence of pro-myelinating astrocytic factors (2-4)

Previous data indicated that myelinating oligodendrocytes cannot only be derived from parenchymal OPCs, but may also come from subependymal neural stem cells located adjacent to the ventricular wall [21, 61, 78]. These ependymal cells present astrocytic features [22] and form a quiescent stem cell reservoir that can give rise to astrocytes and oligodendrocytes after spinal cord injury in mice [4]. We observed an upregulation of the NSC marker nestin as well as an increase in cell proliferation in the SVZ 3/4 days after the osmotic insult. OPCs stemming from SVZ-derived NSCs were further characterized by the co-expression of nestin and PDGFRα. Nestin-positive/PDGFRα-positive cells were not detected in control animals, but only in lesioned tissue. Their density was substantially increased between days 3 and 4 after osmotic stress, decreasing again afterwards. Due to their main proliferation time span beginning after the application period, they  were widely negative for BrdU. Lineage tracing experiments in LPS-induced lesions in rodents revealed that the majority of myelinating oligodendrocytes was derived from parenchymal OPCs rather than subependymal cells [4]. It has been proposed that local NG2-positive OPCs provide a rapid response to acute demyelination, whereas the delayed response of the SVZ mainly serves to fill up the parenchymal OPC pool [82]. Since in our experiments, NG2-positive OPCs in late lesion stages were mostly negative for BrdU, this might also be true for our model. Nevertheless, in our experimental paradigm, actively myelinating as well as mature oligodendrocytes repopulating the lesion were mostly not derived from parenchymal NG2 glia. Therefore, we propose that SVZ-derived oligodendrocytes in striatal ODS lesions not only give rise to OPCs that replace the tissue residents, but also form actively myelinating and consecutively, mature oligodendrocytes. It should be noted that the subventricular zone of the fourth ventricle, which is located close to the dorsal pons and thus may play a role in the repair of demyelinated lesions, did not show an upregulation of nestin in the human CPM patients studied here. In humans, nestin expression in the SVZ seems to be restricted to fetal stages and childhood [20]. Subependymal cells in the SVZ possess stem cell characteristics, giving rise to “fresh” OPCs, whereas parenchymal OPCs of the perilesion tissue are also able to refill the intralesional OPC population but must undergo multiple rounds of self-renewal and accumulate age-related deficiencies. This may presuppose the limited capacity of the adult human brain to regenerate, as compared to rodents, as well as the decline with age (reviewed in: [97]). The proposed dual mechanism leading to the remyelination of astrocyte-depleted experimental ODS lesions is summarized in Fig. 8.

Cortical astrocytes have demonstrated the ability to produce neurospheres in response to injury [14]. In early stages of experimental ODS lesions, few Olig2-positive cells with few short GFAP positive processes could be detected in the mostly astrocyte-free lesion. These cells might represent an intermediate between NSCs (which have astrocytic features) and OPCs. A contribution for trans-differentiated astrocytes to the remyelination of ODS lesions can thus not be excluded.

## Conclusions

In the present study we demonstrate efficient remyelination of experimental ODS lesions and attempts at remyelination of human CPM lesions with primary astrocytopathy [59, 72, 79, 83]. Early astrocyte-depleted lesions were rapidly repopulated with OPCs that differentiated to express BCAS1, but presented a non-myelinating morphology. Upon reappearance of astrocytes, BCAS1-positive cells started to remyelinate the lesion, implying that functional astrocytes are a requisite for remyelination. Our data further indicate that parenchymal as well as stem cell-derived OPCs are participating in the formation of myelinating oligodendrocytes. In experimental striatal, but not in human pontine lesions, neural stem cells (NSCs) located in the SVZ contributed to lesion repair.

The paradigm of osmotic demyelination clearly reveals the contribution of different OPC populations to efficient myelin regeneration. It also points to the significant contribution of fully functional astrocytes to remyelination. Future research will be needed to fully understand the complex interplay between different glial cells. Understanding the cellular and molecular mechanisms of remyelination, including the signals attracting OPCs, the cues leading to efficient internode formation, and the time frame in which astrocyte-derived signals are indispensable, will help tackle the unresolved scientific problem of inefficient myelin repair in major human demyelinating diseases such as MS.

## Supplementary Information

**Additional file 1: Tables S1, S2.** Table S1: Patient data. Table S2: Criteria for staging of human CPM lesions.

**Additional file 2: Fig. S1.** Longitudinal MRI of ODS lesion progression and repair. T2-weighted images (top row) from the same rat are presented at five time points: 0 (baseline), 1, 7, 14, and 21 days post-correction (dpc). The depicted slice is located approximately 2 mm rostrally to the crossing of the anterior commissure. From 7 dpc onwards, bilateral lesions appeared in the ventral striatum (arrows). The lesion location was confirmed by LFB/PAS histochemistry. Regions-of-interest (ROIs) were defined within the lesion area as well as in a normal-appearing area of the dorsal striatum. The lesions can also be seen on parameter maps derived from myelin water imaging and diffusion tensor imaging (bottom image). The within-ROI means of the shown parameters are plotted across time.

**Additional file 3: Fig. S2.** Astrocytic ODS lesion repair. **a** Quantification of GFAP + astrocytes during ODS lesion evolution (one dot represents one animal, mean ± SEM, one-way ANOVA and Tukey’s multiple comparison, *p < 0.05,***p < 0.001). **b** Immunofluorescence double labeling with anti-GFAP and anti-Olig2 antibodies. GFAP = red, Olig2 = green, DAPI = blue, magnification x400, scale bar represents 20 µm.

**Additional file 4: Fig. S3.** Frequency of mature oligodendrocytes over time indicates a directed lesion repair. Heat maps demonstrate the frequency of TPPP/p25+ mature oligodendrocytes in the striatum for representative animals of each time point. Dashed red lines indicate the lesion border. The SVZ (indicated by a dashed purple line) is used to create distance polygons, as exemplarily shown for 3/4dpc (middle graph). For each animal, the frequency of TPPP/p25+ cells is plotted across the distance to the SVZ (right graph). Distances of the inner lesion borders to the SVZ are indicated by a stroke. A bold line indicates the mean for all animals and the shaded area indicates 95% confidence intervals. Lower scheme indicates the typical location of the SVZ and the lesion border in one hemisphere.

**Additional file 5: Fig. S4.** Remyelination in the grey and white matter. Immunofluorescence triple labeling with antibodies against TPPP/p25, CD68 and MBP. Arrowheads mark CD68+ activated microglia/macrophages. TPPP/p25 = red, CD68 = green, MBP = white, DAPI = blue, magnification × 200, scale bar indicates 25 µm.

**Additional file 6: Fig. S5.** Histopathological characteristics of human CPM lesions. Representative microphotographs of typical early, intermediate and late stage CPM lesions. Top row: anti-AQP4 IHC, magnification × 100, scale bar represents 200 µm; middle row: anti-GFAP IHC double labeled with LFB/PAS, magnification × 100, scale bar represents 200 µm; bottom row: anti-KiM1P IHC double labeled with LFB, magnification x200, scale bar represents 100 µm.

**Additional file 7: Fig. S6.** Neural progenitor cells in humans are present in the fetal subventricular zone of the fourth ventricle. Nestin+ cells are abundant in the cell layers adjacent to the fourth ventricle of a fetus (left), but absent in an adult CPM patient (right). Magnification × 400, scale bar represents 50 µm.

## Data Availability

The datasets used and analyzed during the current study are available from the corresponding author on reasonable request.

## Abbreviations

APC: Adenomatous polyposis coli
BCAS1: Breast carcinoma-amplified sequence 1
BrdU: Bromodeoxyuridine
CPM: Central pontine myelinolysis
dpc: Days post-correction
EPM: Extrapontine myelinolysis
FA: Fractional anisotropy
IHC: Immunohistochemistry
LIF: Leukemia inhibitory factor
MAG: Myelin-associated glycoprotein
MRI: Magnetic resonance imaging
MS: Multiple sclerosis
MWF: Myelin water fraction
NMO: Neuromyelitis optica
NSCs: Neural stem cells
ODS: Osmotic demyelinating syndrome
OPCs: Oligodendrocyte precursor cells
PML: Progressive multifocal leukoencephalopathy
RD: Radial diffusivity
SVZ: Subventricular zone
T2IEW: T2 relaxation times for myelin water and intra/extracellular water
TUNEL: Terminal deoxynucleotidyl transferase dUTP nick-end labeling

## References

[1] Adams RD, Victor M, Mancall EL (1959). Central pontine myelinolysis: a hitherto undescribed disease occurring in alcoholic and malnourished patients. AMA Arch Neurol Psychiatry.

[2] Allnoch L, Baumgärtner W, Hansmann F (2019). Impact of astrocyte depletion upon inflammation and demyelination in a murine animal model of multiple sclerosis. Int J Mol Sci.

[3] Bannerman P, Hahn A, Soulika A, Gallo V, Pleasure D (2007). Astrogliosis in EAE spinal cord: derivation from radial glia, and relationships to oligodendroglia. Glia.

[4] Barnabé-Heider F, Göritz C, Sabelström H, Takebayashi H, Pfrieger FW, Meletis K, Frisén J (2010). Origin of new glial cells in intact and injured adult spinal cord. Cell Stem Cell.

[5] Basser P, Mattiello J, LeBihan D (1994). MR diffusion tensor spectroscopy and imaging. Biophys J.

[6] Di Bello IC, Dawson MR, Levine JM, Reynolds R (1999). Generation of oligodendroglial progenitors in acute inflammatory demyelinating lesions of the rat brain stem is associated with demyelination rather than inflammation. J Neurocytol.

[7] Besnard F, Perraud F, Sensenbrenner M, Labourdette G (1987). Platelet-derived growth factor is a mitogen for glial but not for neuronal rat brain cells in vitro. Neurosci Lett.

[8] Blakemore WF, Gilson JM, Crang AJ (2003). The presence of astrocytes in areas of demyelination influences remyelination following transplantation of oligodendrocyte progenitors. Exp Neurol.

[9] Bodini B, Veronese M, García-Lorenzo D, Battaglini M, Poirion E, Chardain A, Freeman L, Louapre C, Tchikviladze M, Papeix C, Dollé F, Zalc B, Lubetzki C, Bottlaender M, Turkheimer F, Stankoff B (2016). Dynamic imaging of individual remyelination profiles in multiple sclerosis. Ann Neurol.

[10] Boretius S, Escher A, Dallenga T, Wrzos C, Tammer R, Brück W, Nessler S, Frahm J, Stadelmann C (2012). Assessment of lesion pathology in a new animal model of MS by multiparametric MRI and DTI. Neuroimage.

[11] Bouchat J, Couturier B, Marneffe C, Gankam-Kengne F, Balau B, De Swert K, Brion J-P, Poncelet L, Gilloteaux J, Nicaise C (2018). Regional oligodendrocytopathy and astrocytopathy precede myelin loss and blood–brain barrier disruption in a murine model of osmotic demyelination syndrome. Glia.

[12] Bramow S, Frischer JM, Lassmann H, Koch-Henriksen N, Lucchinetti CF, Sørensen PS, Laursen H (2010). Demyelination versus remyelination in progressive multiple sclerosis. Brain.

[13] Brown RA, Narayanan S, Banwell B, Arnold DL, Network CPDD (2014). Magnetization transfer ratio recovery in new lesions decreases during adolescence in pediatric-onset multiple sclerosis patients. NeuroImage Clin.

[14] Buffo A, Rite I, Tripathi P, Lepier A, Colak D, Horn A-P, Mori T, Götz M (2008). Origin and progeny of reactive gliosis: a source of multipotent cells in the injured brain. Proc Natl Acad Sci.

[15] Cabana J-F, Gu Y, Boudreau M, Levesque IR, Atchia Y, Sled JG, Narayanan S, Arnold DL, Pike GB, Cohen-Adad J, Duval T, Vuong M-T, Stikov N (2015). Quantitative magnetization transfer imaging made easy with qMTLab: software for data simulation, analysis, and visualization. Concepts Magn Reson Part A.

[16] Chang A, Tourtellotte WW, Rudick R, Trapp BD (2002). Premyelinating oligodendrocytes in chronic lesions of multiple sclerosis. N Engl J Med.

[17] Correale J, Farez MF (2015). The role of astrocytes in multiple sclerosis progression. Front Neurol.

[18] Crawford AH, Tripathi RB, Foerster S, McKenzie I, Kougioumtzidou E, Grist M, Richardson WD, Franklin RJM (2016). Pre-existing mature oligodendrocytes do not contribute to remyelination following toxin-induced spinal cord demyelination. Am J Pathol.

[19] Crawford AH, Tripathi RB, Richardson WD, Franklin RJM (2016). Developmental origin of oligodendrocyte lineage cells determines response to demyelination and susceptibility to age-associated functional decline. Cell Rep.

[20] Dahiya S, Lee DY, Gutmann DH (2011). Comparative characterization of the human and mouse third ventricle germinal zones. J Neuropathol Exp Neurol.

[21] Dimou L, Simon C, Kirchhoff F, Takebayashi H, Götz M (2008). Progeny of Olig2-expressing progenitors in the gray and white matter of the adult mouse cerebral cortex. J Neurosci.

[22] Doetsch F, Caillé I, Lim DA, García-Verdugo JM, Alvarez-Buylla A (1999). Subventricular zone astrocytes are neural stem cells in the adult mammalian brain. Cell.

[23] Domingues HS, Portugal CC, Socodato R, Relvas JB (2016). Oligodendrocyte, astrocyte, and microglia crosstalk in myelin development, damage, and repair. Front Cell Dev Biol.

[24] Duncan ID, Radcliff AB, Heidari M, Kidd G, August BK, Wierenga LA (2018). The adult oligodendrocyte can participate in remyelination. Proc Natl Acad Sci.

[25] Fancy SPJ, Zhao C, Franklin RJM (2004). Increased expression of Nkx2.2 and Olig2 identifies reactive oligodendrocyte progenitor cells responding to demyelination in the adult CNS. Mol Cell Neurosci.

[26] Fard MK, van der Meer F, Sánchez P (2017). BCAS1 expression defines a population of early myelinating oligodendrocytes in multiple sclerosis lesions. Sci Transl Med.

[27] Franklin RJM, Crang AJ, Blakemore WF (1991). Transplanted type-1 astrocytes facilitate repair of demyelinating lesions by host oligodendrocytes in adult rat spinal cord. J Neurocytol.

[28] Franklin RJM, Goldman SA (2015). Glia disease and repair—remyelination. Cold Spring Harb Perspect Biol.

[29] Frischer JM, Weigand SD, Guo Y (2015). Clinical and pathological insights into the dynamic nature of the white matter multiple sclerosis plaque. Ann Neurol.

[30] Gankam Kengne F, Nicaise C, Soupart A, Boom A, Schiettecatte J, Pochet R, Brion JP, Decaux G (2011). Astrocytes are an early target in osmotic demyelination syndrome. J Am Soc Nephrol.

[31] Garyfallidis E, Brett M, Amirbekian B, Rokem A, Van Der Walt S, Descoteaux M, Nimmo-Smith I (2014). Dipy, a library for the analysis of diffusion MRI data. Front Neuroinformatics.

[32] Gocht A, Colmant H (1987). Central pontine and extrapontine myelinolysis: a report of 58 cases. Clin Neuropathol.

[33] Gocht A, Löhler J (1990). Changes in glial cell markers in recent and old demyelinated lesions in central pontine myelinolysis. Acta Neuropathol.

[34] Goldschmidt T, Antel J, König FB, Brück W, Kuhlmann T (2009). Remyelination capacity of the MS brain decreases with disease chronicity. Neurology.

[35] Hammond TR, Gadea A, Dupree J, Kerninon C, Nait-Oumesmar B, Aguirre A, Gallo V (2014). Astrocyte-derived endothelin-1 inhibits remyelination through notch activation. Neuron.

[36] Haynes HR, Gallagher PJ, Cordaro A, Likeman M, Love S (2018). A case of chronic asymptomatic central pontine myelinolysis with histological evidence of remyelination. Forensic Sci Med Pathol.

[37] Horner PJ, Power AE, Kempermann G, Kuhn HG, Palmer TD, Winkler J, Thal LJ, Gage FH (2000). Proliferation and differentiation of progenitor cells throughout the intact adult rat spinal cord. J Neurosci.

[38] Ikota H, Iwasaki A, Kawarai M, Nakazato Y (2010). Neuromyelitis optica with intraspinal expansion of Schwann cell remyelination. Neuropathology.

[39] Ishibashi T, Dakin KA, Stevens B, Lee PR, Kozlov SV, Stewart CL, Fields RD (2006). Astrocytes promote myelination in response to electrical impulses. Neuron.

[40] Junker A, Wozniak J, Voigt D, Scheidt U, Antel J, Wegner C, Brück W, Stadelmann C (2020). Extensive subpial cortical demyelination is specific to multiple sclerosis. Brain Pathol.

[41] Keirstead HS, Levine JM, Blakemore WF (1998). Response of the oligodendrocyte progenitor cell population (defined by NG2 labelling) to demyelination of the adult spinal cord. Glia.

[42] Kerr BJ, Patterson PH (2005). Leukemia inhibitory factor promotes oligodendrocyte survival after spinal cord injury. Glia.

[43] Kleinschmidt-DeMasters BK, Norenberg MD (1981). Rapid correction of hyponatremia causes demyelination: relation to central pontine myelinolysis. Science (80-).

[44] Kleinschmidt-DeMasters BK, Norenberg MD (1982). Neuropathologic observations in electrolyte-induced myelinolysis in the rat. J Neuropathol Exp Neurol.

[45] Kleinschmidt-DeMasters BK, Rojiani AM, Filley CM (2006). Central and extrapontine myelinolysis: then…and now. J Neuropathol Exp Neurol.

[46] Korzhevskii DÉ, Lentsman MV, Gilyarov AV, Kirik OV, Vlasov TD (2008). Induction of nestin synthesis in rat brain cells by ischemic damage. Neurosci Behav Physiol.

[47] Kuhlmann T, Miron V, Cuo Q, Wegner C, Antel J, Brück W (2008). Differentiation block of oligodendroglial progenitor cells as a cause for remyelination failure in chronic multiple sclerosis. Brain.

[48] Lennon VA, Wingerchuk DM, Kryzer TJ, Pittock SJ, Lucchinetti CF, Fujihara K, Nakashima I, Weinshenker BG (2004). A serum autoantibody marker of neuromyelitis optica: distinction from multiple sclerosis. Lancet.

[49] Levine JM, Reynolds R (1999). Activation and proliferation of endogenous oligodendrocyte precursor cells during ethidium bromide-induced demyelination. Exp Neurol.

[50] Levine JM, Stincone F, Lee YS (1993). Development and differentiation of glial precursor cells in the rat cerebellum. Glia.

[51] Lien YH, Shapiro JI, Chan L (1991). Study of brain electrolytes and organic osmolytes during correction of chronic hyponatremia. Implications for the pathogenesis of central pontine myelinolysis. J Clin Investig.

[52] Liu Y, Given KS, Owens GP, Macklin WB, Bennett JL (2018). Distinct patterns of glia repair and remyelination in antibody-mediated demyelination models of multiple sclerosis and neuromyelitis optica. Glia.

[53] Louapre C, Papeix C, Lubetzki C, Maillart E (2017). Multiple sclerosis and aging. Geriatr Psychol Neuropsychiatr Vieil.

[54] MacKay A, Whittall K, Adler J, Li D, Paty D, Graeb D (1994). In vivo visualization of myelin water in brain by magnetic resonance. Magn Reson Med.

[55] MacKay AL, Laule C (2016). Magnetic resonance of myelin water: an in vivo marker for myelin. Brain Plast.

[56] Marignier R, Nicolle A, Watrin C, Touret M, Cavagna S, Varrin-Doyer M, Cavillon G, Rogemond V, Confavreux C, Honnorat J, Giraudon P (2010). Oligodendrocytes are damaged by neuromyelitis optica immunoglobulin G via astrocyte injury. Brain.

[57] Masaki K (2015). Early disruption of glial communication via connexin gap junction in multiple sclerosis, Baló’s disease and neuromyelitis optica. Neuropathology.

[58] Mason JL, Toews A, Hostettler JD, Morell P, Suzuki K, Goldman JE, Matsushima GK (2004). Oligodendrocytes and progenitors become progressively depleted within chronically demyelinated lesions. Am J Pathol.

[59] Matsuoka T, Suzuki SO, Iwaki T, Tabira T, Ordinario AT, Kira J (2010). Aquaporin-4 astrocytopathy in Baló’s disease. Acta Neuropathol.

[60] McTigue DM, Wei P, Stokes BT (2001). Proliferation of NG2-positive cells and altered oligodendrocyte numbers in the contused rat spinal cord. J Neurosci.

[61] Meletis K, Barnabé-Heider F, Carlén M, Evergren E, Tomilin N, Shupliakov O, Frisén J (2008). Spinal cord injury reveals multilineage differentiation of ependymal cells. PLoS Biol.

[62] Menn B, Garcia-Verdugo JM, Yaschine C, Gonzalez-Perez O, Rowitch D, Alvarez-Buylla A (2006). Origin of oligodendrocytes in the subventricular zone of the adult brain. J Neurosci.

[63] Merkler D, Boretius S, Stadelmann C, Ernsting T, Michaelis T, Frahm J, Brück W (2005). Multicontrast MRI of remyelination in the central nervous system. NMR Biomed.

[64] Nicaise C, Marneffe C, Bouchat J, Gilloteaux J (2019). Osmotic demyelination: from an oligodendrocyte to an astrocyte perspective. Int J Mol Sci.

[65] Norenberg MD (2010). Central pontine myelinolysis: historical and mechanistic considerations. Metab Brain Dis.

[66] Norenberg MD, Leslie KO, Robertson AS (1982). Association between rise in serum sodium and central pontine myelinolysis. Ann Neurol.

[67] Nutma E, van Gent D, Amor S, Peferoen LAN (2020). Astrocyte and oligodendrocyte cross-talk in the central nervous system. Cells.

[68] O’Brien D, Kroll R, Johnson G, Covert S, Nelson M (1994). Myelinolysis after correction of hyponatremia in two dogs. J Vet Intern Med.

[69] Patani R, Balaratnam M, Vora A, Reynolds R (2007). Remyelination can be extensive in multiple sclerosis despite a long disease course. Neuropathol Appl Neurobiol.

[70] Patrikios P, Stadelmann C, Kutzelnigg A, Rauschka H, Schmidbauer M, Laursen H, Sorensen PS, Brück W, Lucchinetti C, Lassmann H (2006). Remyelination is extensive in a subset of multiple sclerosis patients. Brain.

[71] Ponath G, Park C, Pitt D (2018). The role of astrocytes in multiple sclerosis. Front Immunol.

[72] Popescu BFG, Bunyan RF, Guo Y, Parisi JE, Lennon VA, Lucchinetti CF (2013). Evidence of aquaporin involvement in human central pontine myelinolysis. Acta Neuropathol Commun.

[73] Prineas JW, Barnard RO, Kwon EE, Sharer LR, Cho E-S (1993). Multiple sclerosis: remyelination of nascent lesions. Ann Neurol.

[74] Prineas JW, Lee S (2019). Multiple sclerosis: destruction and regeneration of astrocytes in acute lesions. J Neuropathol Exp Neurol.

[75] Pringle N, Collarini EJ, Mosley MJ, Heldin CH, Westermark B, Richardson WD (1989). PDGF A chain homodimers drive proliferation of bipotential (O-2A) glial progenitor cells in the developing rat optic nerve. EMBO J.

[76] Radzun H, Hansmann M, Heidebrecht H (1991). Detection of a monocyte/macrophage differentiation antigen in routinely processed paraffin-embedded tissues by monoclonal antibody Ki-M1P. Lab Investig.

[77] Redwine JM, Armstrong RC (1998). In vivo proliferation of oligodendrocyte progenitors expressing PDGFαR during early remyelination. J Neurobiol.

[78] Rivers LE, Young KM, Rizzi M, Jamen F, Psachoulia K, Wade A, Kessaris N, Richardson WD (2008). PDGFRA/NG2 glia generate myelinating oligodendrocytes and piriform projection neurons in adult mice. Nat Neurosci.

[79] Roemer SF, Parisi JE, Lennon VA, Benarroch EE, Lassmann H, Bruck W, Mandler RN, Weinshenker BG, Pittock SJ, Wingerchuk DM, Lucchinetti CF (2007). Pattern-specific loss of aquaporin-4 immunoreactivity distinguishes neuromyelitis optica from multiple sclerosis. Brain.

[80] Ruckh JM, Zhao J-W, Shadrach JL, van Wijngaarden P, Rao TN, Wagers AJ, Franklin RJM (2012). Rejuvenation of regeneration in the aging central nervous system. Cell Stem Cell.

[81] Schindelin J, Arganda-Carreras I, Frise E (2012). Fiji: an open-source platform for biological-image analysis. Nat Methods.

[82] Serwanski DR, Rasmussen AL, Brunquell CB, Perkins SS, Nishiyama A (2018). Sequential contribution of parenchymal and neural stem cell-derived oligodendrocyte precursor cells toward remyelination. Neuroglia (Basel, Switzerland).

[83] Sharma R, Fischer M-T, Bauer J, Felts PA, Smith KJ, Misu T, Fujihara K, Bradl M, Lassmann H (2010). Inflammation induced by innate immunity in the central nervous system leads to primary astrocyte dysfunction followed by demyelination. Acta Neuropathol.

[84] Shi J, Marinovich A, Barres BA (1998). Purification and characterization of adult oligodendrocyte precursor cells from the rat optic nerve. J Neurosci.

[85] Shiow LR, Favrais G, Schirmer L, Schang A-L, Cipriani S, Andres C, Wright JN, Nobuta H, Fleiss B, Gressens P, Rowitch DH (2017). Reactive astrocyte COX2-PGE2 production inhibits oligodendrocyte maturation in neonatal white matter injury. Glia.

[86] Silver J, Schwab ME, Popovich PG (2015). Central nervous system regenerative failure: role of oligodendrocytes, astrocytes, and microglia. Cold Spring Harb Perspect Biol.

[87] Singh TD, Fugate JE, Rabinstein AA (2014). Central pontine and extrapontine myelinolysis: a systematic review. Eur J Neurol.

[88] Skripuletz T, Hackstette D, Bauer K, Gudi V, Pul R, Voss E, Berger K, Kipp M, Baumgärtner W, Stangel M (2012). Astrocytes regulate myelin clearance through recruitment of microglia during cuprizone-induced demyelination. Brain.

[89] Song S-K, Yoshino J, Le TQ, Lin S-J, Sun S-W, Cross AH, Armstrong RC (2005). Demyelination increases radial diffusivity in corpus callosum of mouse brain. Neuroimage.

[90] Sosunov A, Olabarria M, Goldman JE (2018). Alexander disease: an astrocytopathy that produces a leukodystrophy. Brain Pathol.

[91] Stadelmann C, Timmler S, Barrantes-Freer A, Simons M (2019). Myelin in the central nervous system: structure, function, and pathology. Physiol Rev.

[92] Starost L, Lindner M, Herold M, Xu YKT (2020). Extrinsic immune cell-derived, but not intrinsic oligodendroglial factors contribute to oligodendroglial differentiation block in multiple sclerosis. Acta Neuropathol.

[93] Sterns RH, Riggs JE, Schochet SS (1986). Osmotic demyelination syndrome following correction of hyponatremia. N Engl J Med.

[94] Sypecka J, Domanska-Janik K (1995). Expression of myelin-specific proteins during development of normal and hypomyelinatedParalytic tremor mutant rabbits. Mol Chem Neuropathol.

[95] Talbott JF, Loy DN, Liu Y, Qiu MS, Bunge MB, Rao MS, Whittemore SR (2005). Endogenous Nkx2.2+/Olig2+ oligodendrocyte precursor cells fail to remyelinate the demyelinated adult rat spinal cord in the absence of astrocytes. Exp Neurol.

[96] Tripathi RB, Rivers LE, Young KM, Jamen F, Richardson WD (2010). NG2 glia generate new oligodendrocytes but few astrocytes in a murine experimental autoimmune encephalomyelitis model of demyelinating disease. J Neurosci.

[97] Tse K-H, Herrup K (2017). DNA damage in the oligodendrocyte lineage and its role in brain aging. Mech Ageing Dev.

[98] Verbalis JG, Drutarosky MD (1988). Adaptation to chronic hypoosmolality in rats. Kidney Int.

[99] Watanabe M, Toyama Y, Nishiyama A (2002). Differentiation of proliferated NG2-positive glial progenitor cells in a remyelinating lesion. J Neurosci Res.

[100] Weber MS, Derfuss T, Metz I, Brück W (2018). Defining distinct features of anti-MOG antibody associated central nervous system demyelination. Ther Adv Neurol Disord.

[101] Weil M-T, Ruhwedel T, Möbius W, Simons M (2017). Intracerebral Injections and Ultrastructural Analysis of High-Pressure Frozen Brain Tissue. Curr Protoc Neurosci.

[102] Wright DG, Laureno R, Victor M (1979). Pontine and extrapontine mylinolysis. Brain.

[103] Wrzos C, Winkler A, Metz I, Kayser DM, Thal DR, Wegner C, Brück W, Nessler S, Bennett JL, Stadelmann C (2014). Early loss of oligodendrocytes in human and experimental neuromyelitis optica lesions. Acta Neuropathol.

[104] Xing YL, Ro PT, Stratton JAS, Chuang BHA, Danne J, Ellis SL, Ng SW, Kilpatrick TJ, Merson TD (2014). Adult neural precursor cells from the subventricular zone contribute significantly to oligodendrocyte regeneration and remyelination. J Neurosci.

[105] Yao X, Su T, Verkman AS (2016). Clobetasol promotes remyelination in a mouse model of neuromyelitis optica. Acta Neuropathol Commun.

[106] Yeung MSY, Djelloul M, Steiner E, Bernard S, Salehpour M, Possnert G, Brundin L, Frisén J (2019). Dynamics of oligodendrocyte generation in multiple sclerosis. Nature.

[107] Zawadzka M, Rivers LE, Fancy SPJ, Zhao C (2010). CNS-resident glial progenitor/stem cells produce schwann cells as well as oligodendrocytes during repair of CNS demyelination. Cell Stem Cell.

