# Supplementary material for: Lack of astrocytes hinders parenchymal oligodendrocyte precursor cells from reaching a myelinating state in osmolyte-induced demyelination
Source: Acta Neuropathol Commun. 2020 Dec 24;8:224. doi: 10.1186/s40478-020-01105-2 (PMC7761156; doi:10.1186/s40478-020-01105-2)
Supplement: Supplementary file 1 — Additional file 1: Tables S1, S2. Table S1: Patient data. Table S2: Criteria for staging of human CPM lesions. [file 40478_2020_1105_MOESM1_ESM.docx]

Table S1: Patient data.

|  | **Age** | **Sex** | **Group** | **Cause of death** | **Predisposing factors** |
| --- | --- | --- | --- | --- | --- |
| 1 | 43 | f | CPM  Early | Pneumonia | History of alcohol abuse, hepatic steatosis |
| 2 | 41 | m | CPM  Early | Pneumonia | History of alcohol abuse |
| 3 | 58 | m | CPM  Early | Cardiovascular failure | History of alcohol abuse, hepatic steatosis, pancreatitis |
| 4 | 57 | m | CPM  Early | Multiple organ failure | HIV infection,  liver transplantation |
| 5 | 32 | f | CPM  Early | Respiratory insufficiency | History of alcohol abuse, hepatic steatosis,  Guillain Barré syndrome |
| 6 | 46 | m | CPM  Intermediate | n.a. | History of alcohol abuse,  liver fibrosis,  hepatic encephalopathy |
| 7 | 50 | m | CPM  Intermediate | Cardiovascular failure | No |
| 8 | 24 | m | CPM  Intermediate | Multiple organ failure | HIV infection,  hepatomegaly |
| 9 | 47 | m | CPM  Intermediate | Respiratory insufficiency | History of alcohol abuse, hepatic steatosis, |
| 10 | 52 | m | CPM  Intermediate | Multiple organ failure | History of alcohol abuse |
| 11 | 40 | f | CPM  Late | Cardiovascular failure | History of alcohol abuse,  frontotemporal dementia |
| 12 | 63 | m | CPM  Late | Cardiovascular failure | History of alcohol abuse,  history of cortical infarct |
| 13 | 46 | f | CPM  Late | n.a. | History of alcohol abuse, liver fibrosis |
| 14 | 36 | f | CPM  Late | Cardiovascular failure | History of alcohol abuse, liver fibrosis,  hepatic encephalopathy |
| 15 | 50 | m | CPM  Late | Pneumonia | History of alcohol abuse, liver fibrosis |
| 16 | 80 | m | CPM  Late | Cardiovascular failure | History of alcohol abuse, liver fibrosis,  chronic pancreatitis |
| 17 | 15 | m | CPM  Late | Liver failure | Hepatitis B infection,  liver transplantation |
| 18 | 72 | f | CPM  Late | Cardiovascular failure | History of alcohol abuse,  polyneuropathy,  optic atrophy |
| 19 | 58 | m | Control | Cardiac arrest | - |
| 20 | 60 | m | Control | Sepsis, multi organ failure | - |
| 21 | 90 | f | Control | Heart failure | - |
| 22 | 57 | f | Control | Sepsis | - |
| 23 | 39 | f | Control | Pulmonary embolism | - |
| 24 | 65 | m | Control | Trauma | - |
| 25 | 38 | f | Control | Breast carcinoma | - |
| 26 | 62 | m | Control | Suicide | - |
| 27 | GW34 | m | Control | Pulmonary hypoplasia | - |

f: female, m: male, n.a: not available, GW: gestational week

Table S2: Criteria for staging of human CPM lesions

|  | **Astrocytes (GFAP, AQP4)** | **Phagocytes (KiM1P)** | **Myelin (LFB)** |
| --- | --- | --- | --- |
| Early | Reduced density  Often bipolar  Mostly AQP4 negative | High density  Foamy | Disrupted |
| Intermediate | Normal/increased density  Few bipolars  Mostly AQP4 negative | Moderate densities  Partially foamy, partially amoeboid | Absent |
| Late | Normal density  Reactive morphology  AQP4 positive | Moderate density  Inactive/ramified morphology | Pale, remyelinated  or absent |
