# Supplementary figures and images for: Lack of astrocytes hinders parenchymal oligodendrocyte precursor cells from reaching a myelinating state in osmolyte-induced demyelination
Source: Acta Neuropathol Commun. 2020 Dec 24;8:224. doi: 10.1186/s40478-020-01105-2 (PMC7761156; doi:10.1186/s40478-020-01105-2)

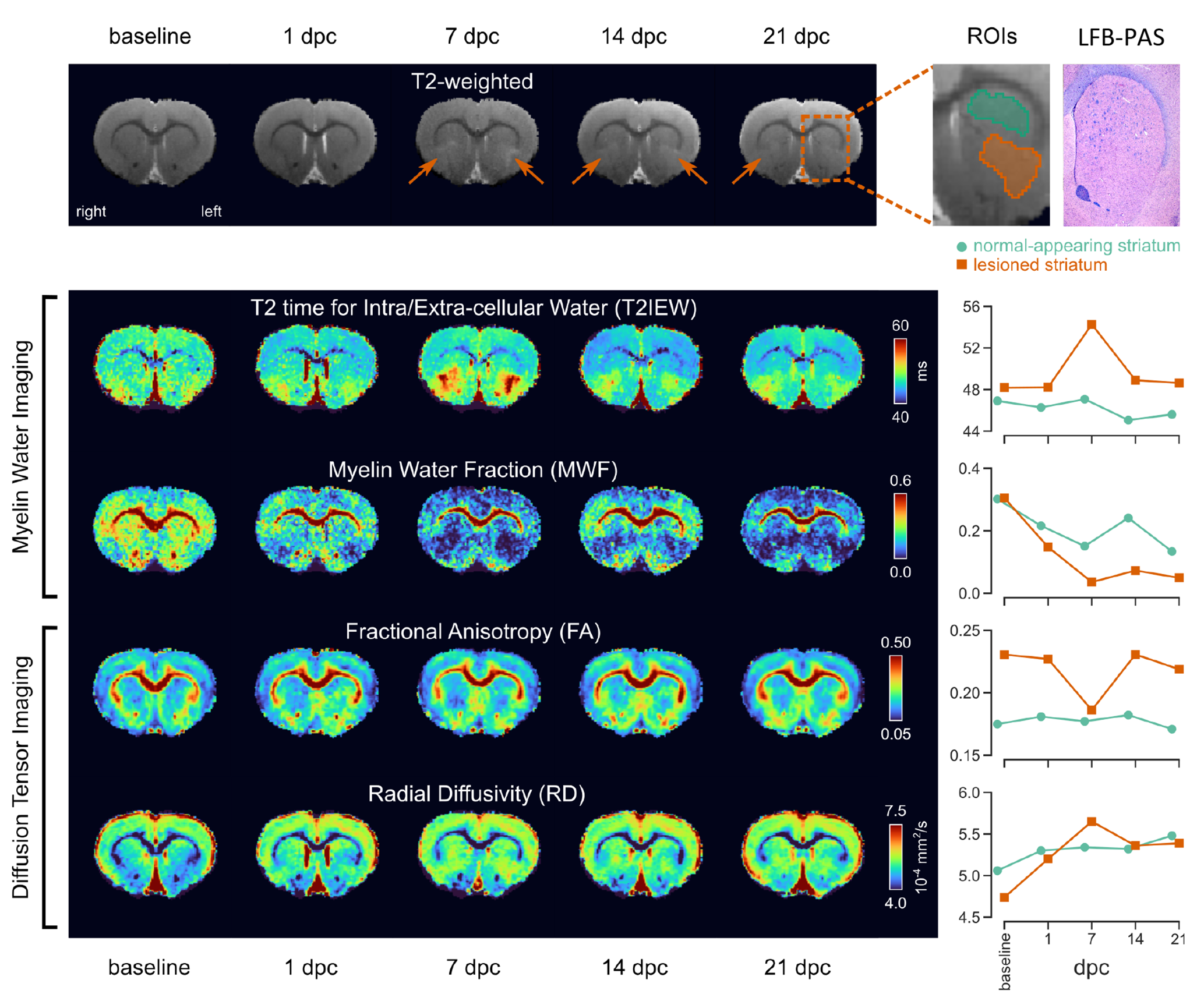

Supplement: Supplementary file 2 — Additional file 2: Fig. S1. Longitudinal MRI of ODS lesion progression and repair. T2-weighted images (top row) from the same rat are presented at five time points: 0 (baseline), 1, 7, 14, and 21 days post-correction (dpc). The depicted slice is located approximately 2 mm rostrally to the crossing of the anterior commissure. From 7 dpc onwards, bilateral lesions appeared in the ventral striatum (arrows). The lesion location was confirmed by LFB/PAS histochemistry. Regions-of-interest (ROIs) were defined within the lesion area as well as in a normal-appearing area of the dorsal striatum. The lesions can also be seen on parameter maps derived from myelin water imaging and diffusion tensor imaging (bottom image). The within-ROI means of the shown parameters are plotted across time. [file 40478_2020_1105_MOESM2_ESM.tif]

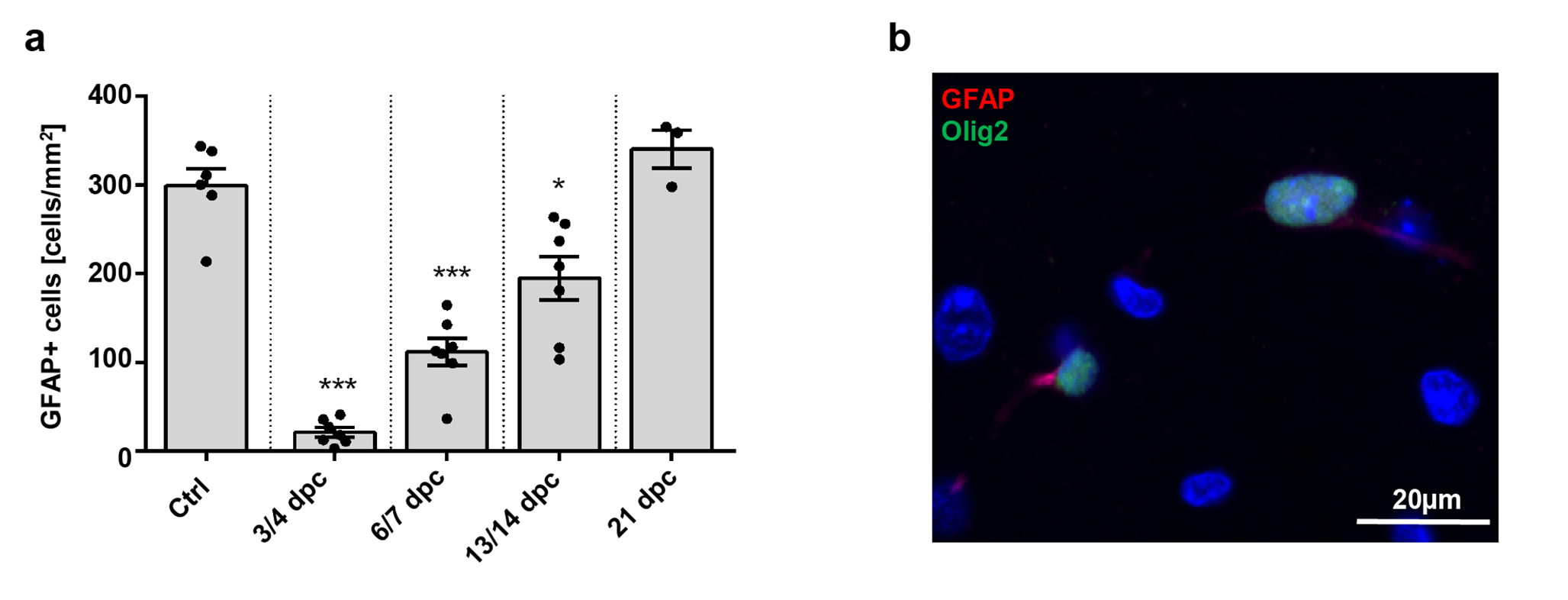

Supplement: Supplementary file 3 — Additional file 3: Fig. S2. Astrocytic ODS lesion repair. a Quantification of GFAP + astrocytes during ODS lesion evolution (one dot represents one animal, mean ± SEM, one-way ANOVA and Tukey’s multiple comparison, *p < 0.05,***p < 0.001). b Immunofluorescence double labeling with anti-GFAP and anti-Olig2 antibodies. GFAP = red, Olig2 = green, DAPI = blue, magnification x400, scale bar represents 20 µm. [file 40478_2020_1105_MOESM3_ESM.tif]

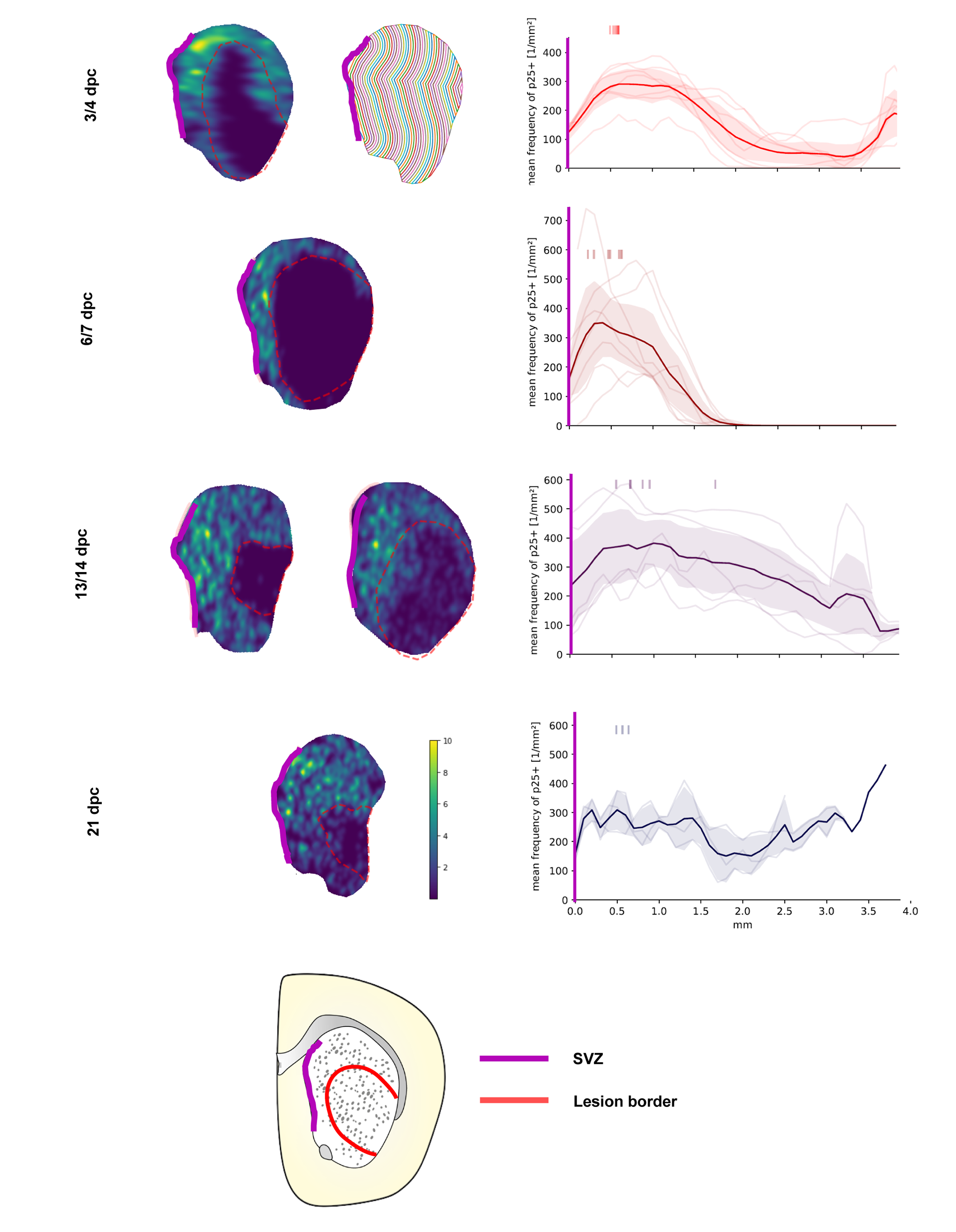

Supplement: Supplementary file 4 — Additional file 4: Fig. S3. Frequency of mature oligodendrocytes over time indicates a directed lesion repair. Heat maps demonstrate the frequency of TPPP/p25+ mature oligodendrocytes in the striatum for representative animals of each time point. Dashed red lines indicate the lesion border. The SVZ (indicated by a dashed purple line) is used to create distance polygons, as exemplarily shown for 3/4dpc (middle graph). For each animal, the frequency of TPPP/p25+ cells is plotted across the distance to the SVZ (right graph). Distances of the inner lesion borders to the SVZ are indicated by a stroke. A bold line indicates the mean for all animals and the shaded area indicates 95% confidence intervals. Lower scheme indicates the typical location of the SVZ and the lesion border in one hemisphere. [file 40478_2020_1105_MOESM4_ESM.tif]

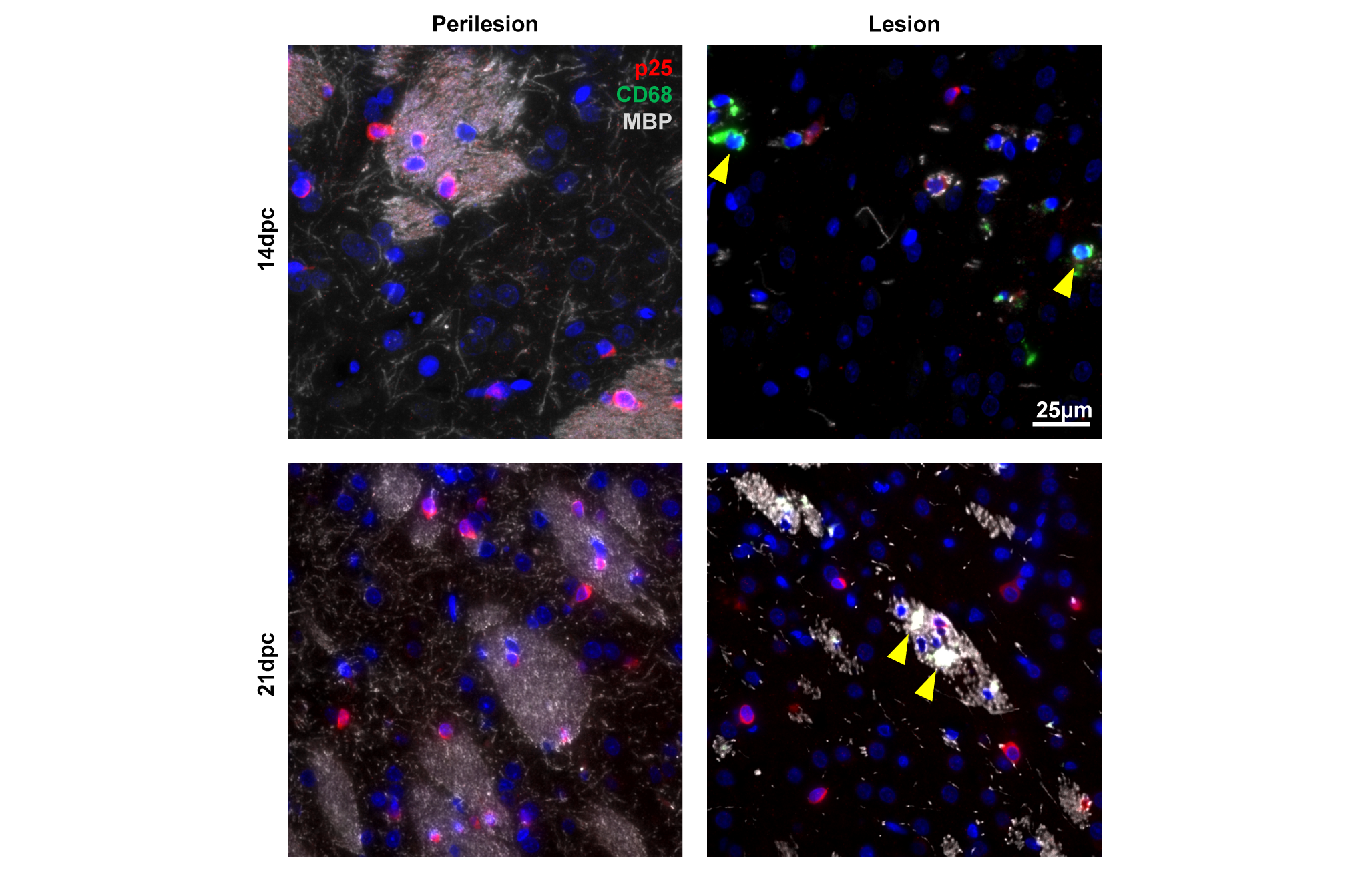

Supplement: Supplementary file 5 — Additional file 5: Fig. S4. Remyelination in the grey and white matter. Immunofluorescence triple labeling with antibodies against TPPP/p25, CD68 and MBP. Arrowheads mark CD68+ activated microglia/macrophages. TPPP/p25 = red, CD68 = green, MBP = white, DAPI = blue, magnification × 200, scale bar indicates 25 µm. [file 40478_2020_1105_MOESM5_ESM.tif]

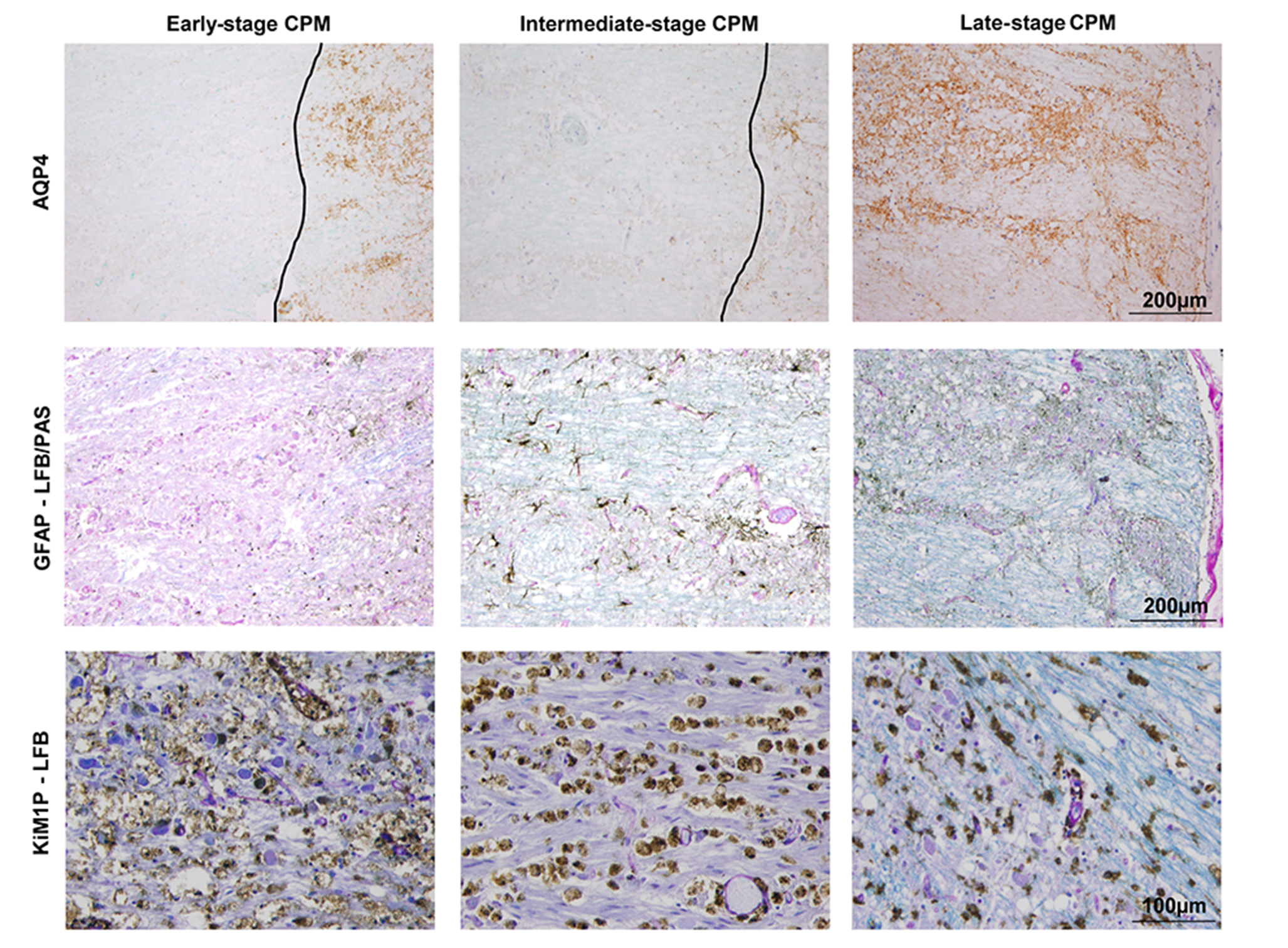

Supplement: Supplementary file 6 — Additional file 6: Fig. S5. Histopathological characteristics of human CPM lesions. Representative microphotographs of typical early, intermediate and late stage CPM lesions. Top row: anti-AQP4 IHC, magnification × 100, scale bar represents 200 µm; middle row: anti-GFAP IHC double labeled with LFB/PAS, magnification × 100, scale bar represents 200 µm; bottom row: anti-KiM1P IHC double labeled with LFB, magnification x200, scale bar represents 100 µm. [file 40478_2020_1105_MOESM6_ESM.tif]

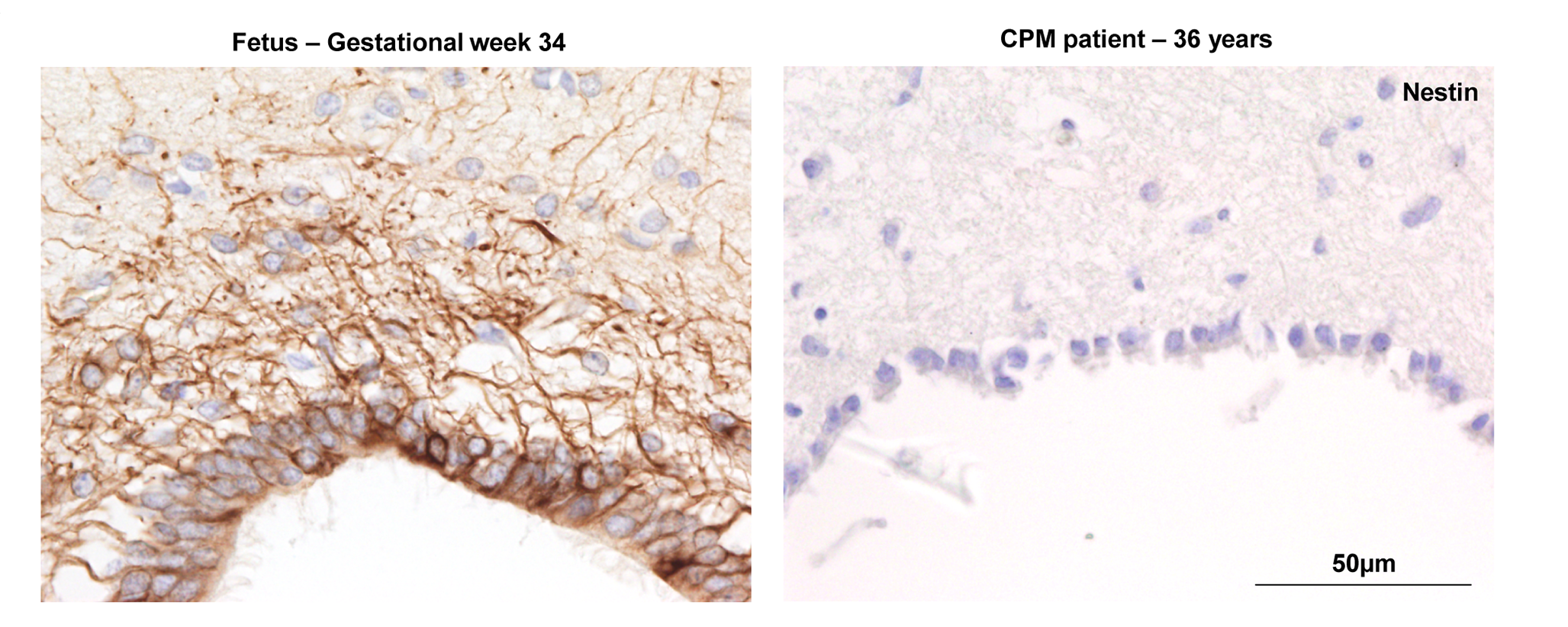

Supplement: Supplementary file 7 — Additional file 7: Fig. S6. Neural progenitor cells in humans are present in the fetal subventricular zone of the fourth ventricle. Nestin+ cells are abundant in the cell layers adjacent to the fourth ventricle of a fetus (left), but absent in an adult CPM patient (right). Magnification × 400, scale bar represents 50 µm. [file 40478_2020_1105_MOESM7_ESM.tif]
